# Comparison of the microbiomes of two drinking water distribution systems—with and without residual chloramine disinfection

**DOI:** 10.1186/s40168-019-0707-5

**Published:** 2019-06-07

**Authors:** Michael B. Waak, Raymond M. Hozalski, Cynthia Hallé, Timothy M. LaPara

**Affiliations:** 1 Department of Civil, Environmental, and Geo-Engineering, University of Minnesota, 500 Pillsbury Dr SE, Minneapolis, 55455 MN USA; 2 Department of Civil and Environmental Engineering, Norwegian University of Science and Technology, S. P. Andersens veg 5, Trondheim, 7491 Norway; 3 BioTechnology Institute, University of Minnesota, 1479 Gortner Ave, Saint Paul, 55108 MN USA

**Keywords:** Drinking water distribution systems, Biofilm bacteria, 16S rRNA gene amplicon sequencing, Residual chloramine disinfection

## Abstract

**Background:**

Residual disinfection is often used to suppress biological growth in drinking water distribution systems (DWDSs), but not without undesirable side effects. In this study, water-main biofilms, drinking water, and bacteria under corrosion tubercles were analyzed from a chloraminated DWDS (USA) and a no-residual DWDS (Norway). Using quantitative real-time PCR, we quantified bacterial 16S rRNA genes and ammonia monooxygenase genes (*amoA*) of *Nitrosomonas oligotropha* and ammonia-oxidizing archaea—organisms that may contribute to chloramine loss. PCR-amplified 16S rRNA genes were sequenced to assess community taxa and diversity.

**Results:**

The chloraminated DWDS had lower biofilm biomass (*P*=1×10^−6^) but higher *N. oligotropha*-like *amoA* genes (*P*=2×10^−7^) than the no-residual DWDS (medians =4.7×10^4^ and 1.1×10^3^*amoA* copies cm^−2^, chloraminated and no residual, respectively); archaeal *amoA* genes were only detected in the no-residual DWDS (median =2.8×10^4^ copies cm^−2^). Unlike the no-residual DWDS, biofilms in the chloraminated DWDS had lower within-sample diversity than the corresponding drinking water (*P*<1×10^−4^). Chloramine was also associated with biofilms dominated by the genera, *Mycobacterium* and *Nitrosomonas* (≤91.7*%* and ≤39.6*%* of sequences, respectively). Under-tubercle communities from both systems contained corrosion-associated taxa, especially *Desulfovibrio* spp. (≤98.4*%* of sequences).

**Conclusions:**

Although residual chloramine appeared to decrease biofilm biomass and alpha diversity as intended, it selected for environmental mycobacteria and *Nitrosomonas oligotropha*—taxa that may pose water quality challenges. Drinking water contained common freshwater plankton and did not resemble corresponding biofilm communities in either DWDS; monitoring of tap water alone may therefore miss significant constituents of the DWDS microbiome. Corrosion-associated *Desulfovibrio* spp. were observed under tubercles in both systems but were particularly dominant in the chloraminated DWDS, possibly due to the addition of sulfate from the coagulant alum.

**Electronic supplementary material:**

The online version of this article (10.1186/s40168-019-0707-5) contains supplementary material, which is available to authorized users.

## Background

Due to a high surface area-to-volume ratio, biofilms comprise a higher fraction of total bacterial biomass than suspended cells in drinking water distribution mains [1, 2]. There is evidence that biofilm bacteria may significantly decrease water quality, increase corrosion of infrastructure, and prolong survival of waterborne pathogens [3–5]. Despite these findings, the influence of water-main biofilms on tap water microbial communities is poorly understood, primarily due to the difficulty of accessing buried water mains to sample resident biofilms.

There is considerable interest in the extent to which residual disinfectants reduce biofilm formation and suppress problematic organisms in full-scale drinking water distribution systems (DWDSs). In the USA, a residual disinfectant, such as free chlorine (HOCl/OCl ^−^) or chloramines (primarily monochloramine, NH_2_Cl), is used to limit undesirable biofilm growth on water-main surfaces and suppress bacteria levels in the bulk water. Recently, chloramine has gained some popularity over free chlorine as a residual disinfectant, largely because of the reduced formation of halogenated disinfection byproducts but possibly also due to its selective reactivity, better penetrating biofilm extracellular polymeric substances [6, 7]. The selective pressure exerted by a disinfectant, however, may create unique communities comprised of resilient microbes [8, 9]. Furthermore, the ammonia (NH_3_/N$\mathrm {H}_{4}^{+}$) released during chloramine decay may stimulate growth of ammonia-oxidizing bacteria (AOB) or ammonia-oxidizing archaea (AOA) [10]. The nitrite (N$\mathrm {O}_{2}^{-}$) produced by AOB or AOA may accelerate chloramine decay, because nitrite readily reacts with chloramine [11].

Due to its various drawbacks, alternatives to residual disinfection have been adopted in many parts of the world, particularly in many European countries [12, 13]. These alternatives include removal of assimilable organic carbon and aggressive maintenance of distribution infrastructure [14, 15]. Unfortunately, regardless of the approach used, water-main biofilms are inevitable. The question remains which scenario—the presence or absence of a residual disinfectant in the DWDS—generally results in a less problematic microbiome.

Herein, we compare the results from an investigation of the microbiomes in two full-scale systems, one DWDS in the USA that maintains a chloramine residual and another in Norway that intentionally has very low or no residual disinfectant in the distributed water. Water mains and drinking water were collected from the two systems. Interior surfaces of the water mains were scraped to collect biofilms and material under corrosion features (i.e., tubercles). Drinking water samples were filtered to obtain suspended cells. After DNA extraction, 16S rRNA gene amplicons from biofilms, under tubercle, and drinking water were sequenced to assess bacterial community composition and diversity. In addition, concentrations of total bacteria, AOB, and AOA were assessed via quantitative real-time polymerase chain reaction (qPCR) targeting, respectively, bacterial 16S rRNA genes, *Nitrosomonas oligotropha*-like ammonia monooxygenase genes (*amoA*), and archaeal *amoA* genes. We previously reported lower quantities of *Legionella* spp. in the chloraminated DWDS relative to the no-residual DWDS [16]. In the present study, we provide a broader assessment of the microbiomes in these two full-scale distribution systems.

## Materials and methods

### Drinking water distribution systems

The chloraminated system in the USA treats river water with lime softening, recarbonation, alum coagulation, sedimentation, filtration, and chloramine disinfection, with an initial residual of 3.8±0.1 mg L^−1^ as Cl_2_ (mean ± standard deviation). In contrast, the no-residual system withdraws lake water from a depth of 50 m. The water passes through granular marble beds (primarily CaCO_3_) to increase alkalinity and water hardness. Water is disinfected with free chlorine and medium-pressure UV radiation (40 mJ cm^−2^), leaving the treatment plant with an initial residual of 0.08±0.01 mg L^−1^ as Cl_2_.

### Water quality

Total chlorine was measured during water sample collection using a Pocket Colorimeter II with DPD powder pillows (Hach Company, Loveland, CO, USA). During a subsequent campaign, assimilable organic carbon (AOC) was measured throughout the two systems (August 2015 and May 2017 for chloraminated and no-residual systems, respectively) using the *Pseudomonas fluorescens* P-17/*Spirillum* sp. NOX method [17]. The chloraminated utility provided raw water temperatures and treated water pH, total chlorine, hardness, free ammonia (NH$_{3} +{NH}_{4}^{+}$), nitrate (NO$_{3}^{-}$), and orthophosphate (P$\mathrm {O}_{4}^{3-}$) for 2014. Water temperatures, total chlorine, free ammonia, and nitrate were also provided for 13 monitoring sites in the distribution system for 2014. The no-residual utility provided raw water temperatures and treated water pH, total chlorine, hardness, ammonium (NH$_{4}^{+}$), and nitrate for 2014 to 2015. Free ammonia in the no-residual DWDS was estimated from ammonium using pH and temperature, as previously described [18]. In addition to water temperature, orthophosphate and total phosphorus were measured at three sites in the no-residual DWDS in May 2017 using Hach assay number LCK 349. The utilities analyze water quality using methods compliant with either the United States Environmental Protection Agency (chloraminated DWDS) or the Standards Norway (no-residual DWDS).

### Sample collection

#### Water mains

Water mains were cut and removed from the DWDS promptly after flow was turned off (i.e., ≤2 h). Prior to cutting, water-main exteriors were rinsed with chlorine bleach (approximately 400 mg L^−1^ as Cl_2_). Depending on water-main material, either a hydraulic chain cutter (unlined grey cast iron or mortar-lined ductile iron) or handheld chop saw (unlined ductile iron) was used. Drinking water was allowed to evacuate from the mains during removal to minimize contamination. The ends of each section were sealed with clean plastic wrap prior to transport to the laboratory. Three to four interior regions of each water main were gently scraped with a flame-sterilized steel microspatula to sample biofilms (median sample area =1.3 cm^2^; range=0.4 to 8.5 cm^2^). If corrosion tubercles were present in a water main, two to four tubercles were pried up and the underlying surfaces gently scraped to recover solids and associated bacteria (median sample area =1.3 cm^2^; range=0.5 to 5.5 cm^2^). Collected solids were released from the spatula by swirling in 0.5 mL lysis buffer (5% sodium dodecyl sulfate, 120 mM sodium phosphate, pH 8) [19].

In total, nine water mains were collected from nine sites in the chloraminated DWDS (sites C2 to C10), and six water mains were collected from three sites in the no-residual DWDS (sites N5 to N7). Maps of the sample locations are provided in Additional file 1: Figure S1. In the chloraminated DWDS, water mains included either unlined grey cast iron or mortar-lined ductile cast iron, with ages of 40 to 127 years. Mains in the no-residual DWDS were made of either unlined grey or unlined ductile cast iron and had ages of 44 to 109 years. Photographs and descriptions of each water main are provided in Additional file 1: Figure S2. Sites with more than one water main were designated using lower-case letters (e.g., N7*a* and N7*b*).

Sites C7 to C10 may be atypical for the chloraminated DWDS because they have no or low flow during the winter months. More explanation is provided in Additional file 1: Text S1. Biofilm samples from these sites were therefore omitted from statistical comparisons between the two systems; drinking water samples from these sites were included, because water was sampled during normal service.

#### Drinking water

Water was collected either directly from the water main via pitot tubes (chloraminated DWDS) or faucets in nearby buildings (no-residual DWDS). To reduce bacterial contamination from the pitot valves or premise plumbing, metal taps were flame-sterilized and plastic taps were rinsed with chlorine bleach. Taps were flushed free of stagnant water for up to 5 min, and then samples were collected in three to four autoclave-sterilized bottles and transported to the laboratory in a cooler. Each sample was individually vacuum-filtered through a nitrocellulose membrane (diameter = 47 mm; pore size = 0.2 *μ*m; EMD Millipore, Bellerica, MA, USA) within an hour of collection (median filtrate volume = 1000 mL; range = 747 to 1265 mL). Filters were then submerged in 0.5 mL lysis buffer. Unfortunately, due to tap availability and service shutoff, drinking water was not collected at every site. Therefore, water samples were collected from six sites in the chloraminated DWDS (sites C1, C2, C4, C6, C7, and C9) and six sites in the no-residual DWDS (sites N1 to N4, N6 to N7), plus treated water from the treatment plant of that system (site N0; Additional file 1: Figure S1).

#### DNA extraction

To recover DNA from lysis buffer, samples were subjected to three freeze-thaw cycles followed by 70 ^∘^C for 90 min. DNA was extracted using the FastDNA SPIN Kit (MP Biomedicals, Santa Ana, CA, USA) and then stored at − 20 ^∘^C.

### High-throughput 16S rRNA gene sequencing

Bacterial 16S rRNA genes were amplified by polymerase chain reaction (PCR) targeting the V3 region (primers 341F/534R) and sequenced by the University of Minnesota Genomics Center to gather microbial community information [20, 21]. To pre-screen samples for sufficient biomass, 16S rRNA gene copy numbers were determined via qPCR, as previously reported [16]. Samples were processed only if the copy number was at least ten times greater than no-template controls, equivalent to ≥1.3×10^4^ gene copies. Subsequent PCR amplification was performed using the quantification cycle (Cq), plus 5 to 10 cycles, to produce amplicons. Purified products were pooled by equal mass into a single amplicon library prior to paired-end MiSeq sequencing (2×150 bp; Illumina, Inc., San Diego, CA, USA), as previously described [16]. Unprocessed paired-end sequence reads have been deposited in the Sequence Read Archive (accession SRP148989) [22, 23].

Sequence reads were trimmed, filtered, and stitched together using “metagenomics-pipeline” (v1.4) [24]. Operational taxonomic units (OTUs) were determined using subsampled open-reference clustering in QIIME (v1.9.1) [25, 26]. Briefly, OTUs were clustered at 97% similarity using USEARCH 6.1 and then assigned consensus taxonomy using the SILVA reference sequences (release 128) [27–29]. Global singletons and sequences that failed PyNAST alignment were removed [30]. A phylogenetic tree was created with FastTree using representative OTU sequences and rooted using the midpoint [31].

Within-sample (alpha) diversity was evaluated using the Shannon and inverse Simpson indices. These were computed using estimated singleton counts to avoid inflation from spurious singletons [32]. Kruskal-Wallis tests were performed to detect significant differences (i.e., *P*≤0.05), followed by Conover-Iman tests without post hoc adjustment to identify pairwise differences [33]. Library size effects were evaluated using Spearman’s rank correlation, which indicated no significant influence of library size (*P* = 0.72 and 0.97 for Shannon and Simpson, respectively; Additional file 1: Figure S3). Rarefaction curves indicated that both indices plateaued rapidly and were therefore robust to library size (Additional file 1: Figure S4).

Between-sample (beta) diversity was assessed using generalized UniFrac distances, without normalization of library sizes [34]. Unweighted UniFrac and Bray-Curtis dissimilarity were also computed to assess the robustness of perceived visual trends and clusters. These alternatives are discussed further in Additional file 1: Text S2. Dimensional reduction was performed using principal coordinates analysis (PCoA) via the “ape” package in R software [35, 36]. To assess library size effects, permutational multivariate analysis of variance (PERMANOVA) was used via the *adonis* function in the “vegan” package [37]. Library size had a minor effect (999 permutations; $R^{2}_{\text {adonis}} = 0.03, P_{\text {adonis}} = 0.001$), though two alternative normalization methods—subsampling without replacement and cumulative sum scaling [38, 39]—yielded similar results. All PERMANOVA tests assessing library size effects are summarized in Additional file 1: Table S1.

### Quantitative real-time PCR

qPCR was performed on a CFX Connect Real-Time PCR Detection System (Bio-Rad Laboratories, Inc., Hercules, CA, USA) targeting *Nitrosomonas oligotropha*-like *amoA* and archaeal *amoA* to quantify, respectively, AOB and AOA populations [40, 41]. qPCR reactions (final volume = 25.0 *μ*L) consisted of 12.5 *μ*L Bio-Rad iTaq SYBR Green Supermix with ROX, 25.0 *μ*g bovine serum albumin (Roche Diagonistics, Indianapolis, IN, USA), optimized forward and reverse primer concentrations to reduce primer dimer, 0.5 *μ*L DNA template, and molecular biology-grade water (Sigma-Aldrich, St. Louis, MO, USA). Forward and reverse PCR primers were synthesized by Integrated DNA Technologies, Inc. (Skokie, IL, USA) and are summarized with their thermoprofiles in Additional file 1: Table S2.

Standard curves were generated using serially diluted solutions of either plasmid DNA (*N. oligotropha*-like *amoA* genes) or custom gBlocks gene fragments (archaeal *amoA* genes). Synthesis of the standards is detailed in Additional file 1: Text S3. PCR amplification efficiencies ranged from 93.0 to 99.7% and 94.4 to 96.6% for *N. oligotropha*-like and archaeal *amoA*, respectively. Standard curves are summarized in Additional file 1: Table S3. Specificity of PCR products was confirmed with melt curves. The limit of quantification (LOQ) was defined as the lowest standard to amplify without primer dimer (5 and 130 copies for *N. oligotropha*-like and archaeal *amoA*, respectively).

qPCR of *amoA* genes was only performed for water-main biofilms and drinking water samples. Gene copy numbers for water-main biofilms and drinking water were normalized by sample area or filtrate volume. The method quantification limit (MQL) was calculated for each sample by applying this normalization to the LOQ copy number. qPCR copy numbers for under-tubercle samples are not shown due to the lack of an appropriate normalization parameter, discussed further in Additional file 1: Text S4. Left-censored observations, including non-detects, were substituted to arbitrarily low numbers (i.e., low ranks) prior to rank-based statistical tests. Independent groups were compared using a generalized Wilcoxon test via the R package, “NADA” [42]. Paired data were compared using a modified sign test [43].

## Results

### Water quality

As previously reported, the chloraminated DWDS had high seasonal variation in distributed water temperature (median = 16.1 ^∘^C; range = 1.8 to 34.1 ^∘^C) and was usually warmer than the no-residual DWDS (median = 7.2 ^∘^C; range = 5.2 to 8.5 ^∘^) [16]. AOC was higher in the chloraminated DWDS (ranges = 238 to 343 versus 81 to 109 µg L ^−1^ as acetate-C), as did free ammonia (< 0.02 to 0.97 mg L ^−1^ as N), nitrate (0.36 to 1.73 mg L ^−1^ as N), and phosphorus (0.17 to 0.52 mg L ^−1^ as P). In the no-residual DWDS, free ammonia and phosphorus were consistently below detection (< 0.02 mg L ^−1^ as N and < 0.05 mg L ^−1^ as P, respectively), and nitrate had a range of 0.22 to 0.26 mg L ^−1^ as N. Water generally had greater hardness in the chloraminated DWDS (44 to 93 mg L ^−1^ as CaCO_3_) than water in the no-residual DWDS (36 to 54 mg L ^−1^ as CaCO_3_). The two systems had similar pH (7.5 to 9.5 versus 7.8 to 8.6, chloraminated and no residual, respectively).

### Taxonomic profiles

After processing the sequences, median library size was 74,883 reads (range = 17,041 to 324,640 reads per sample). Sequence clustering yielded 41,815 unique OTUs among all samples. An analysis of dominant genera—defined as any genus comprising at least 5% of sequences in two samples—indicated differing taxonomic profiles between the chloraminated and no-residual systems.

#### Water-main biofilms

**Fig. 1:**
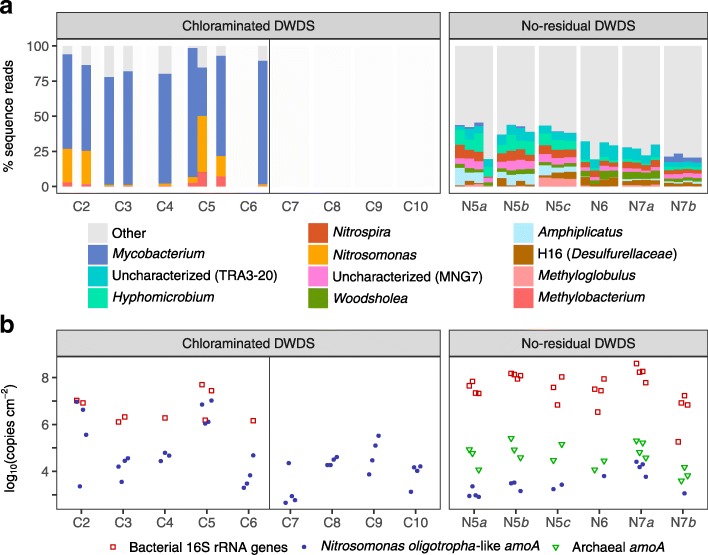
Characterization of water-main biofilms. **a** Taxonomic profiles of dominant genera in biofilms via 16S rRNA gene amplicon sequencing and **b** marker gene concentrations via qPCR. Samples not sequenced due to low 16S rRNA gene copy numbers are represented with empty space

Biofilms were taxonomically and structurally different between the two systems (Fig. 1a). Biofilms in the chloraminated DWDS were unevenly distributed—skewed by three dominant genera: *Mycobacterium* (≤91.7*%* of sequence reads), *Nitrosomonas* (≤39.6*%*), and *Methylobacterium* (≤10.3*%*). In contrast, water-main biofilms in the no-residual DWDS were more evenly distributed and included many taxa: *Hyphomicrobium* (≤13.2*%*), *Amphiplicatus* (≤12.4*%*), *Nitrospira* (≤9.7*%*), *Methyloglobulus* (≤6.4*%*), H16 of family *Desulfurellaceae* (≤6.1*%*), and *Woodsholea* (≤5.9*%*), as well as uncharacterized genera of order TRA3-20 within *Betaproteobacteria* (≤13.0*%*) and family MNG7 within order *Rhizobiales* (≤6.8*%*). Winter-shutoff water mains from the chloraminated DWDS were not assessed due to insufficient biomass.

#### Drinking water

**Fig. 2:**
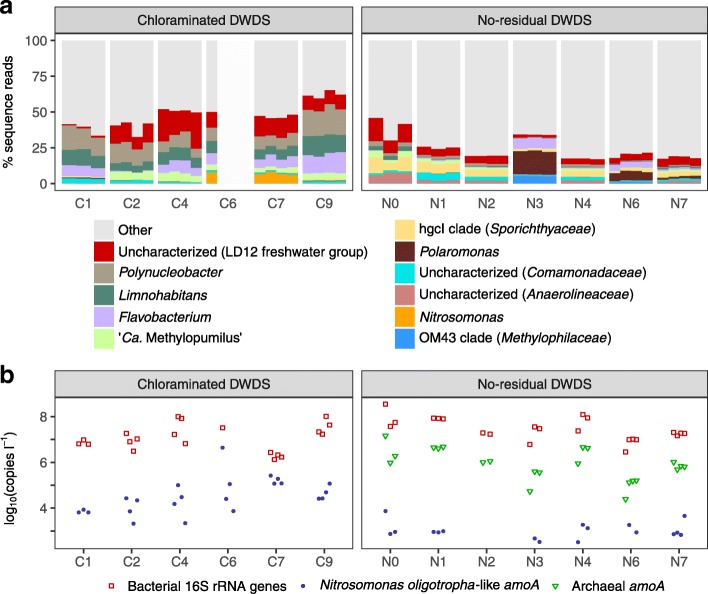
Characterization of drinking water bacteria. **a** Taxonomic profiles of dominant genera in drinking water via 16S rRNA gene amplicon sequencing and **b** gene concentrations via qPCR. Samples not sequenced due to low 16S rRNA gene copy numbers are represented with empty space

In both systems, drinking water samples were comprised of different dominant taxa than the respective biofilms (Fig. 2a). Taxa common to both systems included uncharacterized members of the LD12 freshwater group of *Alphaproteobacteria*, *Polynucleobacter*, *Limnohabitans*, *Flavobacterium*, and “*Candidatus* Methylopumilus.” *Nitrosomonas*-like OTUs were prominent at sites C6 and C7 in the chloraminated DWDS. In the no-residual DWDS, the hgcI clade of family *Sporichthyaceae* and uncharacterized members of families *Comamonadaceae* and *Anaerolineaceae* were also dominant, and *Polaromonas*-like OTUs were detected intermittently (especially sites N3, N6, and N7).

#### Under tubercles

**Fig. 3:**
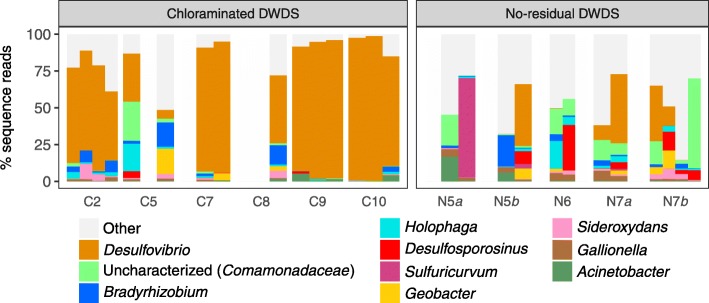
Characterization of under-tubercle bacteria. Taxonomic profiles of dominant genera in under-tubercle communities 16S rRNA gene amplicon sequencing. Samples not sequenced due to low biomass are represented with empty space

*Desulfovibrio*-like OTUs were common under tubercles (Fig. 3), especially in the chloraminated DWDS (≤98.4*%* of sequence reads versus ≤47.0*%* in the no-residual DWDS). Other common taxa included *Desulfosporosinus*, *Holophaga*, *Bradyrhizobium*, *Acinetobacter*, uncharacterized *Comamonadaceae*, *Sideroxydans*, and *Gallionella*.

#### Nitrification-related taxa

AOB encompass two lineages of class *Proteobacteria*: genus *Nitrosococcus* in the gamma subclass and family *Nitrosomonadaceae* in the beta subclass, which includes genera *Nitrosomonas* and *Nitrosospira* [44]. Family *Nitrosomonadaceae*-like OTUs were ubiquitous among biofilm and drinking water samples in both systems (Additional file 1: Figure S5). Genus *Nitrosomonas*, in particular, was dominant in biofilms from the chloraminated DWDS (Fig. 1a), whereas most *Nitrosomonadaceae* in the no-residual DWDS were uncharacterized at genus level. *Nitrosococcus*-like OTUs were not detected.

Nitrite-oxidizing bacteria (NOB), which convert nitrite to nitrate, comprise at least seven genera in four phyla [45]. *Nitrospira*, *Nitrolancea*, *Nitrococcus*, *Nitrotoga*, and *Nitrobacter* have been associated with engineered environments [45]. *Nitrospira*-like OTUs were prominent in the no-residual DWDS but were also detected in the chloraminated DWDS along with *Nitrotoga*- and *Nitrobacter*-like OTUs (Additional file 1: Figure S5). In canonical two-step nitrification, the expected ratio of NOB to AOB is 0.5 [46]. Expected concentrations of NOB and AOB were calculated by normalizing relative abundances of the NOB- and AOB-like OTUs by 16S rRNA gene concentrations (Additional file 1: Figure S6).

### Gene marker concentrations

*N. oligotropha*-like *amoA* and archaeal *amoA* gene concentrations, as well as previously reported values of bacterial 16S rRNA gene concentrations [16], are shown in Figs. 1b and 2b and summarized in Table 1.

**Table 1:** Summary of marker gene concentrations via qPCR

Target	Statistic	Biofilms (copies cm ^−2^)		*P* value ^*†*,*‡*^	Drinking water (copies L ^−1^)		*P* value ^*†*^	Source
		Chloraminated ^*‡*^	No residual		Chloraminated	No residual		
Bacterial 16S rRNA genes	Median	<MQL	4.5×10^7^		7.9×10^6^	2.2×10^7^		
	Min.	<6.5×10^5^	1.8×10^5^	1×10^−6^	<1.3×10^6^	2.8×10^6^	5×10^−3^	[16]
	Max.	5.0×10^7^	4.0×10^8^		1.0×10^8^	3.5×10^8^		
*N. oligotropha*-like *amoA* genes	Median	4.7×10^4^	1.1×10^3^		2.6×10^4^	7.9×10^2^		
	Min.	2.0×10^3^	<3.2×10^2^	2×10^−7^	2.1×10^3^	<2.2×10^2^	5×10^−10^	This study
	Max.	1.0×10^7^	2.6×10^4^		4.4×10^6^	7.4×10^3^		
Archaeal *amoA* genes	Median	<MQL	2.8×10^4^		<MQL	9.2×10^5^		
	Min.	<1.1×10^2^	<1.5×10^3^	4×10^−6^	<2.3×10^2^	2.5×10^4^	3×10^−11^	This study
	Max.	<7.1×10^2^	2.6×10^5^		<5.6×10^2^	1.4×10^7^		

<*MQL*, less than method quantification limit; *min.*, minimum; *max.*, maximum

^*†*^Group-wise comparisons (i.e., chloraminated versus no residual) using generalized Wilcoxon tests

^*‡*^Observations from seasonal shut-off water mains are excluded

#### 16S rRNA genes

Water-main biofilms from the chloraminated DWDS were frequently < MQL and significantly lower (*P*=1×10^−6^) than those in the no-residual DWDS. The median 16S rRNA gene concentration in the chloraminated DWDS was < MQL (MQL range =3.2×10^5^ to 3.2×10^6^ copies cm^−2^), whereas the median was 4.5×10^7^ copies cm^−2^ in the no-residual DWDS. Drinking water concentrations were also significantly lower (*P*=0.005) in the chloraminated DWDS, with a median that was approximately 40% of the median in the no-residual DWDS.

#### Ammonia monooxygenase genes

Compared to the no-residual DWDS, there were higher *N. oligotropha*-like *amoA* concentrations in both the biofilms and drinking water of the chloraminated DWDS (*P*=2×10^−7^ and 5×10^−10^, respectively, using the Wilcoxon test). Archaeal *amoA* genes were not observed in the chloraminated DWDS but were present in the no-residual DWDS and—when compared pair-wise within samples of that system—were significantly higher than *N. oligotropha*-like *amoA* concentrations (*P*=0.019 using the modified sign test). Ratios of *N. oligotropha*-like *amoA* to 16S rRNA genes correlated well to the relative abundances of *Nitrosomonas*-like OTUs (Spearman’s *ρ*=0.95,*P*=3×10^−15^ and *Nitrosomonadaceae*-like OTUs (*ρ*=0.94,*P*=3×10^−14^) in the chloraminated DWDS but not in the no-residual DWDS (*ρ*=− 0.57,*P*=0.008 for *Nitrosomonas* and *ρ*=0.16,*P*=0.5 for *Nitrosomonadaceae*; Additional file 1: Figure S7).

### Alpha diversity

**Fig. 4:**
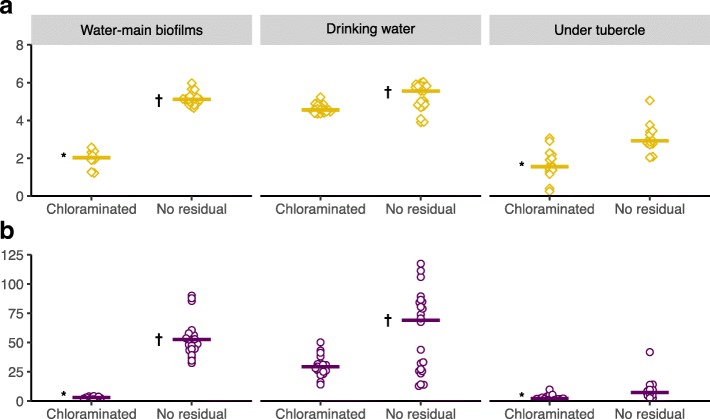
Alpha diversity of water-main biofilms, drinking water, and under tubercle. **a** Shannon index (higher values = greater richness and evenness) and **b** inverse Simpson index (higher values = greater evenness). Shared symbols (e.g., * and *†*) indicate no significant group-wise difference (i.e., *P*>0.05), and bars indicate medians

Shannon and Simpson indices are provided in Fig. 4. Biofilm, drinking water, and under-tubercle samples in the chloraminated DWDS had significantly lower Shannon (*P*≤ 0.0001,<0.0001, and 0.0006, respectively) and Simpson indices (*P*≤ 0.0001,<0.0001, and 0.001) than the corresponding sample groups from the no-residual DWDS. Water-main biofilms in the no-residual DWDS had similar Shannon and Simpson indices to the drinking water samples from that DWDS (*P*=0.8 and 0.3, respectively), while in the chloraminated DWDS, the biofilms and drinking water samples had significantly different Shannon and Simpson indices (*P* ≤ 0.0001 and <0.0001). In the chloraminated DWDS, water-main biofilm alpha diversity index values were not significantly different than the corresponding under tubercle values (*P*=0.6 for both indices). All *P* values are summarized in Additional file 1: Tables S4 and S5.

### Beta diversity

**Fig. 5:**
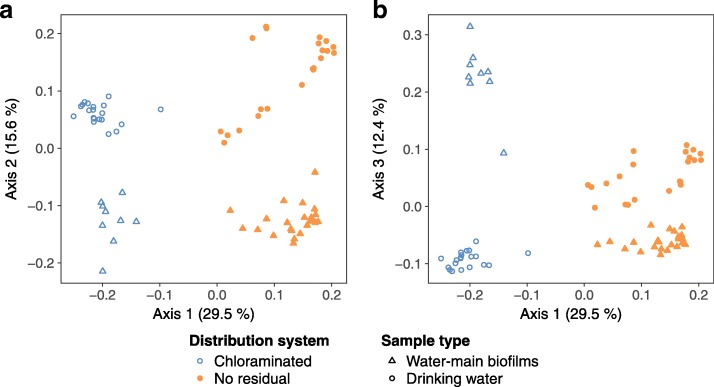
Beta diversity of water-main biofilms and drinking water principal coordinates analysis of the generalized UniFrac distances for water-main biofilms and drinking water from the chloraminated and no-residual drinking water distribution systems: **a** principal axis 2 versus 1 and **b** principal axis 3 versus 1. Percentages = variance explained

Using PCoA of generalized UniFrac distances, samples clustered by distribution system and sample type (Additional file 1: Figure S8). Because biofilms may interact with drinking water (and vice versa), these samples were compared separately from under-tubercle samples, which were isolated environments. Variance among the biofilm and drinking water samples (Fig. 5) was explained primarily by the distribution system of origin (principal axis 1) and then sample type (axes 2 and 3). Under-tubercle samples (6) showed moderate partitioning by distribution system (axis 1), albeit less pronounced than observed in biofilms and drinking water. PCoA of unweighted UniFrac (Additional file 1: Figure S9) and Bray-Curtis dissimilarity (Additional file 1: Figure S10) provided similar visual patterns to generalized UniFrac in all datasets (i.e., all samples, biofilms and drinking water only, and under tubercle only).

**Fig. 6:**
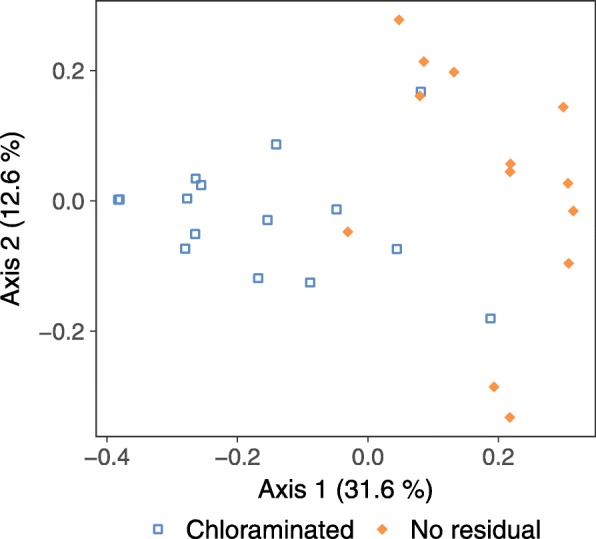
Beta diversity of under-tubercle bacteria. Principal coordinates analysis of the generalized UniFrac distances (principal axis 2 versus 1) for under-tubercle samples from the chloraminated and no-residual drinking water distribution systems. Percentages = variance explained

## Discussion

Bacteria in the DWDS, whether attached to surfaces as biofilms or suspended in the water, are important in that they can have impacts on public health as well as distribution system infrastructure. Detailed characterizations of the microbiomes in full-scale distribution systems, however, are lacking owing to the difficulty of obtaining biofilms from water mains. Drinking water analysis is insufficient, as suspended bacteria may represent < 5% of total biomass in the DWDS microbiome [1, 2]. In this investigation, water and water-main biofilms were sampled from two distribution systems to characterize and compare their microbiomes: one DWDS that maintains a chloramine residual and another without residual disinfectant. The results provide novel information on relationships between biofilms and suspended biomass within a DWDS and suggest possible ecological effects of a residual disinfectant on the DWDS microbiome.

In both systems, drinking water had markedly different taxonomic profiles than the corresponding water-main biofilms. Though the viability of these taxa was not determined, the matrix-specific communities agreed with previous studies and indicated that tap water samples provide an incomplete assessment of the DWDS microbiome [2, 47]. Among drinking water samples, observed taxa are often associated with rivers and lakes—consistent with the water sources of both systems. *Limnohabitans*, *Polynucleobacter*, *Flavobacterium*, “*Candidatus* Methylopumilus,” the hgcI clade, and the LD12 freshwater group are all important bacterial plankton [48–52]. The prevalence of plankton-like taxa in the water, which were not present in corresponding biofilms, suggested separate communities in the water and biofilms—a finding that is not surprising due to the vast differences between suspended and surface-associated microbial lifestyles [53].

Based on the dominant taxa, water-main biofilms appeared to be adapted for nutrient scarcity. This is consistent with a meta-analysis of diverse bacterial communities across distinct habitats that found all exhibited predictable patterns during primary succession—resulting in mature, late-succession communities well-adapted to low-resource conditions [54]. In both systems, taxa that are considered facultative methylotrophs were common in the biofilms. A feature of methylotrophs is their ability to use single-carbon compounds, such as methanol and formate, as sole carbon and energy source [55]. Facultative methylotrophs, including *Mycobacterium*, *Methylobacterium*, and *Hyphomicrobium* spp., have been associated with biofilms in water meters and premise plumbing [55, 56]. In the present study, *Methylobacterium*- and especially *Mycobacterium*-like OTUs were primarily associated with the chloraminated DWDS—consistent with other chloraminated systems [9, 57]—while *Hyphomicrobium*-like OTUs were prevalent in the no-residual DWDS. Our findings were also consistent with a survey of 17 municipal distribution systems that found *Mycobacterium* and *Methylobacterium* spp. were enriched in drinking water with high chloramine concentrations, whereas *Hyphomicrobium* spp.—among other genera—were more abundant in water with low concentrations of free chlorine [58]. Methylotrophy is common among environmental bacteria, and the variety of potential substrates these organisms can utilize may make them particularly well-adapted for low-nutrient environments, such as drinking water distribution systems [55, 59].

In the chloraminated DWDS, the mutual presence of mycobacteria and methylobacteria, which compete for a similar ecological niche, was interesting, because methylobacteria may outcompete mycobacteria when disinfectant residuals are diminished [60]. Notably, *Methylobacterium*-like OTUs, along with *Nitrosomonas*, were most prominent in two heavily tuberculated water mains (sites C2 and C5). It is unclear whether this was related to the tuberculation. Certain corrosion products, however, can impart a chloramine demand, resulting in ammonia production [61].

*Mycobacterium*-like OTUs were far more abundant in the chloraminated DWDS—similar to another chloraminated DWDS in the USA [9]. Environmental mycobacteria include opportunistic pathogens, such as *Mycobacterium avium* complex [62]. We previously reported an ostensible association between the presence of residual chloramine and lower quantities of *Legionella* spp. in the water-main biofilms [16]. Thus, chloramine-induced enrichment of *Mycobacterium* spp., if pathogenic, may indicate a trade-off and potential dilemma for chloraminated systems. Pathogenic and non-pathogenic types of environmental mycobacteria, however, cannot be distinguished using 16S rRNA genes [63]. Many species have been associated with drinking water supplies, likely because mycobacterial cell membranes are waxy and afford them tolerance to chlorine and chloramine [64].

The presence of residual chloramine appeared to further differentiate biofilms from the bulk drinking water. In the chloraminated DWDS, the lower richness and evenness of biofilms (relative to the corresponding drinking water) was consistent with selection by chloramine disinfection. It was therefore not surprising that the dominant taxa were also taxa well-adapted to the presence of chloramine: chloramine-tolerant *Mycobacterium* spp. and ammonia-seeking *Nitrosomonas* spp. In contrast, drinking water and biofilms in the no-residual DWDS—though comprised of mutually distinct populations—shared similar overall richness and evenness.

Environmental conditions, including seasonally warmer water temperatures and consistently higher assimilable nutrients (i.e., AOC, ammonia, nitrate, phosphorus), appeared more favorable to microbial growth in the chloraminated DWDS [16]. Yet, bacterial biomass was significantly lower in the drinking water and especially the water-main biofilms of that system compared to the no-residual DWDS [16]. The lower biomass in the chloraminated DWDS was attributed to bacterial inactivation by the residual chloramine. Lower species richness due to chloramine, however, may also contribute to a decrease in total biomass. A decrease in species richness may be accompanied by lower metabolic diversity, meaning potential substrates that sustain growth may be under-utilized. Notably, the taxonomy of 16S rRNA gene sequences in the no-residual DWDS biofilms hinted at the presence of diverse chemotrophic lifestyles, including methane oxidation (*Methanoglobulus* spp.), ammonia or nitrite oxidation (family *Nitrosomonadaceae* and *Nitrospira* spp.), and sulfur reduction (family *Desulfurellaceae*) [44, 45, 65, 66]. Though we did not assess metabolic expression, the higher diversity of taxa observed in the no-residual DWDS could enable more diverse utilization of substrates.

The high relative abundances of *Nitrosomonas*-like OTUs in the chloraminated DWDS biofilms may pose challenges for the management of nitrification in that system. *Nitrosomonas* spp. are well-known AOB. In canonical two-step nitrification, AOB or AOA produce nitrite, which is further oxidized to nitrate—either biologically by NOB or abiotically by various possible reaction pathways. Notably, nitrite and chloramine react abiotically to produce additional ammonia as well as nitrate [67]. The subsequent ammonia may further stimulate AOB or AOA. The rapid loss of chloramine due to microbial activity, known as biologically accelerated chloramine decay, is a major challenge for water utilities because a diminished residual may enable bacterial growth [68]. Furthermore, nitrite and nitrate are both toxic, with maximum contaminant limits in the USA of 1.0 mg L ^−1^ and 10.0 mg L ^−1^ as N, respectively.

There was a deficiency of NOB in the chloraminated DWDS relative to AOB. The expected biomass yield of NOB is approximately 50% of AOB, giving a theoretical NOB to AOB ratio of 0.5 when NOB have uncontested access to nitrite [46]. Yet, there was an orders-of-magnitude surplus of AOB-like OTUs present in the biofilms relative to expected NOB abundances. The reaction between chloramine and nitrite is rapid compared to the metabolic oxidation of nitrite via NOB [67, 69]. Thus, the chloramine demand exerted by nitrite likewise exhausts nitrite availability, diminishing NOB populations. In contrast, the NOB to AOB ratios in the no-residual DWDS were more consistent with two-step nitrification, though less informative for that system due to the additional presence of AOA (approximately 10^3^ times higher than AOB).

Though free ammonia was not detected in the drinking water of the no-residual DWDS, there were nitrification-relevant taxa present in both the drinking water and the biofilms of that system. The higher concentration of archaeal *amoA* genes over *N. oligotropha*-like *amoA* genes was consistent with an ammonia-deficient environment [70]. In drinking water from the Netherlands with low ammonia (< 0.05 mg L ^−1^ as NH_4_), AOA ranged from 10^6^ to 10^7^ copies L ^−1^ of *amoA* and outnumbered AOB-like *amoA* by a factor of 10^3^ [71]. *Nitrospira*-like OTUs were also abundant in the no-residual DWDS and may have included comammox *Nitrospira*, as they are efficient at utilizing ammonia in nutrient-poor environments and have been previously observed in drinking water systems [72, 73]. The prominence of AOA concurrent to *Nitrospira* has been seen in deep, oligotrophic freshwater lakes—similar to the water supply source of the no-residual DWDS—and may be related to the carbon fixation strategies of these organisms [74]. Overall, the apparent diversity of nitrifiers in the no-residual DWDS (i.e., AOA, AOB, and NOB) was in contrast to the *Nitrosomonas*-dominated biofilms in the chloraminated DWDS and mirrored the broader observations of richness and evenness in the two systems. Though advantageous to AOB as an ammonia source, chloramine may nonetheless only benefit chloramine-tolerant AOB—in this case, *Nitrosomonas* spp. with *N. oligotropha*-like *amoA* genes. This was consistent with previous reports that chloramine selects for *N. oligotropha* [10].

Despite the stark contrasts in drinking water and biofilm communities from the two distribution systems, the under-tubercle communities from the two systems were taxonomically similar. Under-tubercle samples from the chloraminated DWDS were, however, significantly less diverse than those from the no-residual DWDS. We had assumed that the under-tubercle communities from the chloraminated DWDS had been sufficiently shielded from residual chloramine, due to their physical separation from the bulk water and the reactivity of chloramine with corrosion products [61]. Chloramine may indirectly, however, lower the diversity under tubercles by selectively culling primary colonizers, which presumably originate from the chloraminated water. The diversity and taxonomic composition of water-main bacterial communities, however, may be significantly influenced by pipe material [75], which we unfortunately cannot address due to our limited sample quantities.

*Desulfovibrio*-like OTUs were commonly the most dominant taxonomic group in the chloraminated DWDS, which was consistent with the tubercles of another chloraminated DWDS [9]. Sulfate-reducing *Desulfovibrio* spp. are commonly associated with microbiologically influenced corrosion and corroded, iron-rich environments [4, 9, 75, 76]. The electron donor for these organisms is unknown but could be H_2_ from reduction of protons via iron corrosion. The higher relative abundances of *Desulfovibrio*-like OTUs in the chloraminated DWDS may be attributed to sulfate in the treated water (27.6±3.2 mg L ^−1^ as SO$_{4}^{2-}$) from coagulation with aluminum sulfate (Al _2_(SO_4_)_3_). In contrast, no sulfate was added to water in the no-residual DWDS during treatment, and *Desulfovibrio*-like OTUs were prominent in only a few samples.

## Conclusion

In addition to nutrient scarcity, chloramine is likely a fundamental factor influencing water-main biofilms in a chloraminated drinking water distribution system. Richness and evenness were significantly lower in the biofilms of the chloraminated DWDS relative to the corresponding water samples. In contrast, there was no significant difference in within-sample diversity between water-main biofilms and drinking water from the no-residual DWDS. Total biomass was also significantly lower in the chloraminated DWDS, despite seasonally higher water temperatures and higher AOC, ammonia, nitrate, and phosphorus. Additional investigation of water-main biofilms from full-scale systems is necessary, however, to determine whether the observations for the chloraminated and no-residual systems in this study extend to other similar systems. Nonetheless, the ostensible shift in taxonomic composition toward mycobacteria and *Nitrosomonas oligotropha*-like AOB suggested that certain taxa tolerate residual chloramine and may even benefit from it. These taxa may pose problems: some mycobacteria are potential pathogens and AOB may contribute to accelerated chloramine decay. In addition, corrosion-associated *Desulfovibrio* spp. were common underneath corrosion tubercles in both systems, suggesting that microbiological activity may contribute to cast-iron corrosion regardless of whether a disinfectant residual is maintained in the bulk drinking water. Though the chloramine appeared to work as intended—reducing biomass and preventing most taxa present in the bulk water from integrating into the biofilms—the remaining biofilm taxa may pose management challenges. It therefore remains unclear whether the effectiveness of chloramine outweighs the potential problems arising from its selection of specific, well-adapted taxa in the DWDS microbiome.

## Additional file

Additional file 1 Supplemental Information (PDF). Supplemental text and references explaining water mains from winter-shutoff sites (Text S1), library-size normalization for beta metrics (Text S2), synthesis of qPCR standards (Text S3), and qPCR of under-tubercle samples (Text S4); supplementary figures include sample collection locations (Figure S1), water-main photos (Figure S2), alpha diversity versus library size (Figure S3), rarefaction analysis of alpha diversity (Figure S4), nitrifier-like OTUs (Figure S5), NOB to AOB ratios (Figure S6), AOB-like OTUs versus *amoA* to 16S rRNA gene ratios (Figure S7), beta diversity of all samples using PCoA of generalized UniFrac (Figure S8), beta diversity using PCoA of unweighted UniFrac (Figure S9), and beta diversity using PCoA of Bray-Curtis dissimilarity (Figure S10); supplemental tables include PERMANOVA tests assessing library size on beta diversity (Table S1), PCR primer sequences and thermoprofiles (Table S2), qPCR summary statistics (Table S3), between-group comparisons using Conover-Iman tests of Shannon indices (Table S4), and between-group comparisons using Conover-Iman tests of inverse Simpson indices (Table S5). (PDF 3386 kb)

## Data Availability

The datasets generated and/or analyzed during the current study are available in the Sequence Read Archive under accession number SRP148989 (https://www.ncbi.nlm.nih.gov/sra) and BioProject PRJNA433427 [22]. Any other datasets used and/or analyzed during the current study are available from the corresponding author on reasonable request.

## References

[1] Flemming HC, Percival SL, Walker JT (2002). Contamination potential of biofilms in water distribution systems. Wa Sci Technol.

[2] Liu G, Bakker GL, Li S, Vreeburg JHG, Verberk JQJC, Medema GJ (2014). Pyrosequencing reveals bacterial communities in unchlorinated drinking water distribution system: an integral study of bulk water, suspended solids, loose deposits, and pipe wall biofilm. Environ Sci Technol.

[3] Liu G, Verberk JQJC, van Dijk JC (2013). Bacteriology of drinking water distribution systems: an integral and multidimensional review. Appl Microbiol Biotechnol.

[4] Beech IB, Biocorrosion SunnerJ (2004). Towards understanding interactions between biofilms and metals. Curr Opin Biotech.

[5] Wingender J, Flemming HC (2011). Biofilms in drinking water and their role as reservoir for pathogens. Int J Hyg Envir Heal.

[6] Stalter D, O’Malley E, von Gunten U, Escher BI (2016). Fingerprinting the reactive toxicity pathways of 50 drinking water disinfection by-products. Water Res.

[7] Xue Z, Lee WH, Coburn KM, Seo Y (2014). Selective reactivity of monochloramine with extracellular matrix components affects the disinfection of biofilm and detached clusters. Environ Sci Technol.

[8] Chiao TH, Clancy TM, Pinto A, Xi C, Raskin L (2014). Differential resistance of drinking water bacterial populations to monochloramine disinfection. Environ Sci Technol.

[9] Gomez-Smith CK, LaPara TM, Hozalski RM (2015). Sulfate reducing bacteria and mycobacteria dominate the biofilm communities in a chloraminated drinking water distribution system. Environ Sci Technol.

[10] Regan JM, Harrington GW, Baribeau H, De Leon R, Noguera DR (2003). Diversity of nitrifying bacteria in full-scale chloraminated distribution systems. Water Res.

[11] American Water Works Association (2002). Nitrification.

[12] van der Kooij D, van Lieverloo JHM, Schellart JA, Hiemstra P (1999). Distributing drinking water without disinfectant: highest achievement or height of folly. J Water SRT–Aqua.

[13] Rosario-Ortiz F, Rose J, Speight V, von Gunten U, Schnoor J (2016). How do you like your tap water. Science.

[14] Prest EI, Hammes F, van Loosdrecht MCM, Vrouwenvelder JS (2016). Biological stability of drinking water: controlling factors, methods, and challenges. Front Microbiol.

[15] Douterelo I, Husband S, Loza V, Boxall J (2016). Dynamics of biofilm regrowth in drinking water distribution systems. Appl Environ Microb.

[16] Waak MB, LaPara TM, Hallé C, Hozalski RM (2018). Occurrence of Legionella spp. in water-main biofilms from two drinking water distribution systems. Environ Sci Technol.

[17] Rice EW, Baird RB, Eaton AD, Clesceri LS (2012). Standard methods for the examination of water and wastewater. 22nd ed.

[18] Emerson K, Russo RC, Lund RE, Thurston RV (1975). Aqueous ammonia equilibrium calculations: effect of pH and temperature. J Fish Res Board Can.

[19] LaPara TM, Nakatsu CH, Pantea L, Alleman JE (2000). Phylogenetic analysis of bacterial communities in mesophilic and thermophilic bioreactors treating pharmaceutical wastewater. Appl Environ Microbiol.

[20] Muyzer G, de Waal EC, Uitterlinden AG (1993). Profiling of complex microbial-populations by denaturing gradient gel-electrophoresis analysis of polymerase chain reaction-amplified genes coding for 16S rRNA. Appl Environ Microb.

[21] Bartram AK, Lynch MDJ, Stearns JC, Moreno-Hagelsieb G, Neufeld JD (2011). Generation of multimillion-sequence 16S rRNA gene libraries from complex microbial communities by assembling paired-end Illumina reads. Appl Environ Microb.

[22] Waak MB, LaPara TM, Hallé C, Hozalski RM. Drinking water 16S rRNA gene raw sequence reads. Seq Read Arch. 2018. accession number SRP148989; BioProject PRJNA433427. https://www.ncbi.nlm.nih.gov/bioproject/PRJNA433427.

[23] Leinonen R, Sugawara H, Shumway M (2010). The sequence read archive. Nucleic Acids Res.

[24] Garbe JR, Gould TJ, Gohl DM, Knights D, Beckman KB (2017). Metagenomics-pipeline.

[25] Caporaso JG, Kuczynski J, Stombaugh J, Bittinger K, Bushman FD, Costello EK (2010). QIIME allows analysis of high-throughput community sequencing data. Nat Methods.

[26] Rideout JR, He Y, Navas-Molina JA, Walters WA, Ursell LK, Gibbons SM (2014). Subsampled open-reference clustering creates consistent, comprehensive OTU definitions and scales to billions of sequences. PeerJ.

[27] Edgar RC (2010). Search and clustering orders of magnitude faster than BLAST. Bioinformatics.

[28] Quast C, Pruesse E, Yilmaz P, Gerken J, Schweer T, Yarza P (2012). The SILVA ribosomal RNA gene database project: improved data processing and web-based tools. Nucleic Acids Res.

[29] Yilmaz P, Parfrey LW, Yarza P, Gerken J, Pruesse E, Quast C (2013). The SILVA and “All-species Living Tree Project (LTP)” taxonomic frameworks. Nucleic Acids Res.

[30] Caporaso JG, Bittinger K, Bushman FD, DeSantis TZ, Andersen GL, Knight R (2010). PyNAST: a flexible tool for aligning sequences to a template alignment. Bioinformatics.

[31] Price MN, Dehal PS, Arkin AP (2010). FastTree 2 –Approximately maximum-likelihood trees for large alignments. PLoS ONE.

[32] Chiu CH, Chao A (2016). Estimating and comparing microbial diversity in the presence of sequencing errors. PeerJ.

[33] Moran MD (2003). Arguments for rejecting the sequential Bonferroni in ecological studies. Oikos.

[34] Chen J, Bittinger K, Charlson ES, Hoffmann C, Lewis J, Wu GD (2012). Associating microbiome composition with environmental covariates using generalized UniFrac distances. Bioinformatics.

[35] Paradis E, Claude J, Strimmer K. APE (2004). Analyses of phylogenetics and evolution in R language. Bioinformatics.

[36] R Core Team (2014). R: a language and environment for statistical computing.

[37] Oksanen J, Blanchet FG, Friendly M, Kindt R, Legendre P, McGlinn D, et al.vegan: community ecology package; 2018. https://CRAN.R-project.org/package=vegan.

[38] Weiss S, Xu ZZ, Peddada S, Amir A, Bittinger K, Gonzalez A (2017). Normalization and microbial differential abundance strategies depend upon data characteristics. Microbiome.

[39] Paulson JN, Stine OC, Bravo HC, Pop M (2013). Differential abundance analysis for microbial marker-gene surveys. Nature.

[40] Harms G, Layton AC, Dionisi HM, Gregory IR, Garrett VM, Hawkins SA (2003). Real-time PCR quantification of nitrifying bacteria in a municipal wastewater treatment plant. Environ Sci Technol.

[41] Meinhardt KA, Bertagnolli A, Pannu MW, Strand SE, Brown SL, Stahl DA (2015). Evaluation of revised polymerase chain reaction primers for more inclusive quantification of ammonia-oxidizing archaea and bacteria. Env Microbiol Rep.

[42] Lopaka L. NADA: Nondetects And Data Analysis for environmental data; 2017. https://CRAN.R-project.org/package=NADA.

[43] Fong DYT, Kwan CW, Lam KF, Lam KSL (2003). Use of the sign test for the median in the presence of ties. Am Stat.

[44] Purkhold U, Pommerening-Röser A, Juretschko S, Schmid MC, Koops HP, Wagner M (2000). Phylogeny of all recognized species of ammonia oxidizers based on comparative 16S rRNA and amoA sequence analysis: implications for molecular diversity surveys. Appl Environ Microb.

[45] Daims H, Lücker S, Wagner M (2016). A new perspective on microbes formerly known as nitrite-oxidizing bacteria. Trends Microbiol.

[46] Winkler MKH, Bassin JP, Kleerebezem R, Sorokin DY, van Loosdrecht MCM (2012). Unravelling the reasons for disproportion in the ratio of AOB and NOB in aerobic granular sludge. Appl Microbiol Biotechnol.

[47] Pinto AJ, Xi C, Raskin L (2012). Bacterial community structure in the drinking water microbiome is governed by filtration processes. Environ Sci Technol.

[48] Zwart G, Crump BC, Kamst-van Agterveld MP, Hagen F, Han SK (2002). Typical freshwater bacteria: an analysis of available 16S rRNA gene sequences from plankton of lakes and rivers. Aquat Microb Ecol.

[49] Watanabe K, Komatsu N, Ishii Y, Negishi M (2009). Effective isolation of bacterioplankton genus Polynucleobacter from freshwater environments grown on photochemically degraded dissolved organic matter. FEMS Microbiol Ecol.

[50] Salcher MM, Pernthaler J, Posch T (2011). Seasonal bloom dynamics and ecophysiology of the freshwater sister clade of SAR11 bacteria ‘that rule the waves’ (LD12). ISME J.

[51] Llirós M, García-Armisen T, Anzil A, Leporcq B, Pigneur LM, Inceoğlu Ö (2014). Bacterial community composition in three freshwater reservoirs of different alkalinity and trophic status. PLoS ONE.

[52] Salcher MM, Neuenschwander SM, Posch T, Pernthaler J (2015). The ecology of pelagic freshwater methylotrophs assessed by a high-resolution monitoring and isolation campaign. ISME J.

[53] Donlan RM (2002). Biofilms: microbial life on surfaces. Emerg Infect Dis.

[54] Ortiz-Álvarez R, Fierer N (2018). de los Ríos A, Casamayor EO, Barberán A. Consistent changes in the taxonomic structure and functional attributes of bacterial communities during primary succession. ISME J.

[55] Liu R, Zhu J, Yu Z, Joshi D, Zhang H, Lin W (2014). Molecular analysis of long-term biofilm formation on PVC and cast iron surfaces in drinking water distribution system. J Environ Sci.

[56] Ling F, Hwang C, LeChevallier MW, Andersen GL, Liu WT (2016). Core-satellite populations and seasonality of water meter biofilms in a metropolitan drinking water distribution system. ISME J.

[57] Kelly JJ, Minalt N, Culotti A, Pryor M, Packman A (2014). Temporal variations in the abundance and composition of biofilm communities colonizing drinking water distribution pipes. PLoS ONE.

[58] Stanish LF, Hull NM, Robertson CE, Harris JK, Stevens MJ, Spear JR (2016). Factors influencing bacterial diversity and community composition in municipal drinking waters in the Ohio River Basin, USA. PLoS ONE.

[59] Chistoserdova L, Kalyuzhnaya MG (2018). Current trends in methylotrophy. Trends Microbiol.

[60] Falkinham IIIJO, Williams MD, Kwait R, Lande L (2016). *Methylobacterium* spp. as an indicator for the presence or absence of Mycobacterium spp.. Int J Myco.

[61] Woolschlager JE, Rittmann BE, Piriou P, Schwartz B (2001). Developing an effective strategy to control nitrifier growth using the Comprehensive Disinfection and Water Quality Model (CDWQ). World Water and Environmental Resources Congress 2001.

[62] Falkinham IIIJO. (2002). Nontuberculous mycobacteria in the environment. Clin Chest Med.

[63] Beye M, Fahsi N, Raoult D, Fournier PE (2018). Careful use of 16S rRNA gene sequence similarity values for the identification of Mycobacterium species. New Microbe and New Infect.

[64] Luh J, Tong N, Raskin L, Mariñas BJ (2008). Inactivation of Mycobacterium avium with monochloramine. Environ Sci Technol.

[65] Deutzmann JS, Hoppert M, Schink B (2014). Characterization and phylogeny of a novel methanotroph, Methyloglobulus morosus gen. nov., spec. nov. Syst Appl Microbiol.

[66] Rosenberg E, DeLong EF, Lory S, Stackebrandt E, Thompson F (2014). The Family Desulfurellaceae.

[67] Vikesland PJ, Ozekin K, Valentine RL (2001). Monochloramine decay in model and distribution system waters. Water Res.

[68] Bal Krishna KC, Sathasivan A, Sarker DC (2012). Evidence of soluble microbial products accelerating chloramine decay in nitrifying bulk water samples. Water Res.

[69] Nowka B, Daims H, Spieck E (2015). Comparison of oxidation kinetics of nitrite-oxidizing bacteria: nitrite availability as a key factor in niche differentiation. Appl Environ Microb.

[70] Martens-Habbena W, Berube PM, Urakawa H, de la Torre JR, Stahl DA (2009). Ammonia oxidation kinetics determine niche separation of nitrifying Archaea and Bacteria. Nature.

[71] Wielen PWJJvd VoostS (2009). Ammonia-oxidizing bacteria and archaea in groundwater treatment and drinking water distribution systems. Appl Environ Microbiol.

[72] Kits KD, Sedlacek CJ, Lebedeva EV, Han P, Bulaev A, Pjevac P (2017). Kinetic analysis of a complete nitrifier reveals an oligotrophic lifestyle. Nature.

[73] Wang Y, Ma L, Mao Y, Jiang X, Xia Y, Yu K (2017). Comammox in drinking water systems. Water Res.

[74] Alfreider A, Grimus V, Luger M, Ekblad A, Salcher MM, Summerer M (2018). Autotrophic carbon fixation strategies used by nitrifying prokaryotes in freshwater lakes. FEMS Microbiol Ecol.

[75] Ren H, Wang W, Liu Y, Liu S, Lou L, Cheng D (2015). Pyrosequencing analysis of bacterial communities in biofilms from different pipe materials in a city drinking water distribution system of East China. Appl Microbiol Biotechnol.

[76] Bolton N, Critchley M, Fabien R, Cromar N, Fallowfield H (2010). Microbially influenced corrosion of galvanized steel pipes in aerobic water systems. J Appl Microbiol.

