# Supplementary material for: Comparison of the microbiomes of two drinking water distribution systems—with and without residual chloramine disinfection
Source: Microbiome. 2019 Jun 7;7:87. doi: 10.1186/s40168-019-0707-5 (PMC6556008; doi:10.1186/s40168-019-0707-5)
Supplement: Supplementary file 1 — Supplemental Information (PDF). Supplemental text and references explaining water mains from winter-shutoff sites (Text S1), library-size normalization for beta metrics (Text S2), synthesis of qPCR standards (Text S3), and qPCR of under-tubercle samples (Text S4); supplementary figures include sample collection locations (Figure S1), water-main photos (Figure S2), alpha diversity versus library size (Figure S3), rarefaction analysis of alpha diversity (Figure S4), nitrifier-like OTUs (Figure S5), NOB to AOB ratios (Figure S6), AOB-like OTUs versus amoA to 16S rRNA gene ratios (Figure S7), beta diversity of all samples using PCoA of generalized UniFrac (Figure S8), beta diversity using PCoA of unweighted UniFrac (Figure S9), and beta diversity using PCoA of Bray-Curtis dissimilarity (Figure S10); supplemental tables include PERMANOVA tests assessing library size on beta diversity (Table S1), PCR primer sequences and thermoprofiles (Table S2), qPCR summary statistics (Table S3), between-group comparisons using Conover-Iman tests of Shannon indices (Table S4), and between-group comparisons using Conover-Iman tests of inverse Simpson indices (Table S5). (PDF 3386 kb) [file 40168_2019_707_MOESM1_ESM.pdf]

SUPPLEMENTARY INFORMATION

Comparison of the microbiomes of two drinking water distribution systems — with and without residual chloramine disinfection

Michael B. Waak,[1,2] Raymond M. Hozalski,[1,3] Cynthia Hallé,[2] and Timothy M. LaPara[1,3,*]

[1] Department of Civil, Environmental, and Geo-Engineering, University of Minnesota, 500 Pillsbury Dr. SE, Minneapolis, Minnesota 55455, U.S.A.
[2] Department of Civil and Environmental Engineering, Norwegian University of Science and Technology, S.P. Andersens veg 5, Trondheim NO-7491, Norway
[3] BioTechnology Institute, University of Minnesota, 1479 Gortner Ave., Saint Paul, Minnesota 55108, U.S.A.

[*] Corresponding author; e-mail: lapar001@umn.edu

18 pages: 4 supplemental texts (Texts S1 to S4); 10 supplemental figures (Figs. S1 to S10); 5 supplemental tables (Tables S1 to S5)

Supplemental Text

S1 Water mains from winter-shutoff sites

Though collected during normal operation, the water mains at these sites are shutoff annually during the cold-weather months due to freezing concerns; though technically still operational and full of drinking water, these mains lie adjacent to gate-valves that are closed to completely halt flow. Water in the main on the opposite side of each gate-valve is drained to prevent ice formation and expansion damage. These dead ends are likely stagnant for months-long periods every year.

S2 Library-size normalization for beta metrics

Beta diversity was assessed using the generalized UniFrac distances and checked using the unweighted UniFrac distances and Bray-Curtis dissimilarity as alternatives. Depending on the metric, read counts were normalized to account for the uneven library sizes [S1]. No normalization was performed for generalized UniFrac (i.e. original counts were used) [S2]. For unweighted UniFrac, counts were equally subsampled to the lowest count of any sample [S3]. Bray-Curtis dissimilarity was calculated using read counts normalized with cumulative sum scaling [S4].

S3 Synthesis of qPCR standards

Standards were created using either plasmid DNA (*Nitrosomonas oligotropha*-like *amoA* genes) or custom gBlocks gene fragments (archaeal *amoA* genes). Plasmid DNA was prepared from PCR amplification using positive controls, followed by ligation with pGEM-T Easy cloning vectors (Promega, Madison, WI, USA) and transformation into *Escherichia coli* JM109. After purification with the QIAprep Spin Miniprep Kit (QIAGEN, Hilden, Germany), plasmid DNA was stained with Hoechst 33258 dye and quantified on a TD-700 fluorometer (Turner Designs, Sunnyvale, CA, USA) using calf thymus DNA as a standard. For archaeal *amoA* gene standards, custom gBlocks gene fragments were synthesized by Integrated DNA Technologies using a reference fragment from *Nitrosopumilus maritimus* (GenBank accession HM345610; `https://www.ncbi.nlm.nih.gov/genbank`).

S4 qPCR of under-tubercle samples

The 16S rRNA gene copy numbers for under-tubercle samples are not shown due to the lack of an appropriate normalization parameter. Surface area was deemed inappropriate as there appeared to be substantial differences in the masses of corrosion solids recovered from under different tubercles. Dry mass of corrosion solids may have been a suitable parameter, but unfortunately, masses were not quantified. Furthermore, AOB and AOA were not considered relevant for the under-tubercle communities, which were ostensibly oxygen-deficient, so qPCR was not performed targeting either *amoA* gene target.

Supplemental References

[S1] McMurdie PJ, Holmes S. Waste Not, Want Not: Why rarefying microbiome data is inadmissible. *PLoS Comput Biol* 2014; **10**: e1003531.

[S2] Chen J, Bittinger K, Charlson ES, Hoffmann C, Lewis J, Wu GD, *et al*. Associating microbiome composition with environmental covariates using generalized UniFrac distances. *Bioinformatics* 2012; **28**: 2106–2113.

[S3] Weiss S, Xu ZZ, Peddada S, Amir A, Bittinger K, Gonzalez A, *et al*. Normalization and microbial differential abundance strategies depend upon data characteristics. *Microbiome* 2017; **5**: 27.

[S4] Paulson JN, Stine OC, Bravo HC, Pop M. Differential abundance analysis for microbial marker-gene surveys. *Nature* 2013; **10**: 1200–1202.

[S5] Bartram AK, Lynch MDJ, Stearns JC, Moreno-Hagelsieb G, Neufeld JD. Generation of multimillion-sequence 16S rRNA gene libraries from complex microbial communities by assembling paired-end Illumina reads. *Appl Environ Microb* 2011; **77**: 3846–3852.

[S6] Harms G, Layton AC, Dionisi HM, Gregory IR, Garrett VM, Hawkins SA, *et al*. Real-time PCR quantification of nitrifying bacteria in a municipal wastewater treatment plant. *Environ Sci Technol* 2003; **37**: 343–351.

[S7] Meinhardt KA, Bertagnolli A, Pannu MW, Strand SE, Brown SL, Stahl DA. Evaluation of revised polymerase chain reaction primers for more inclusive quantification of ammonia-oxidizing archaea and bacteria. *Env Microbiol Rep* 2015; **7**: 354–363.

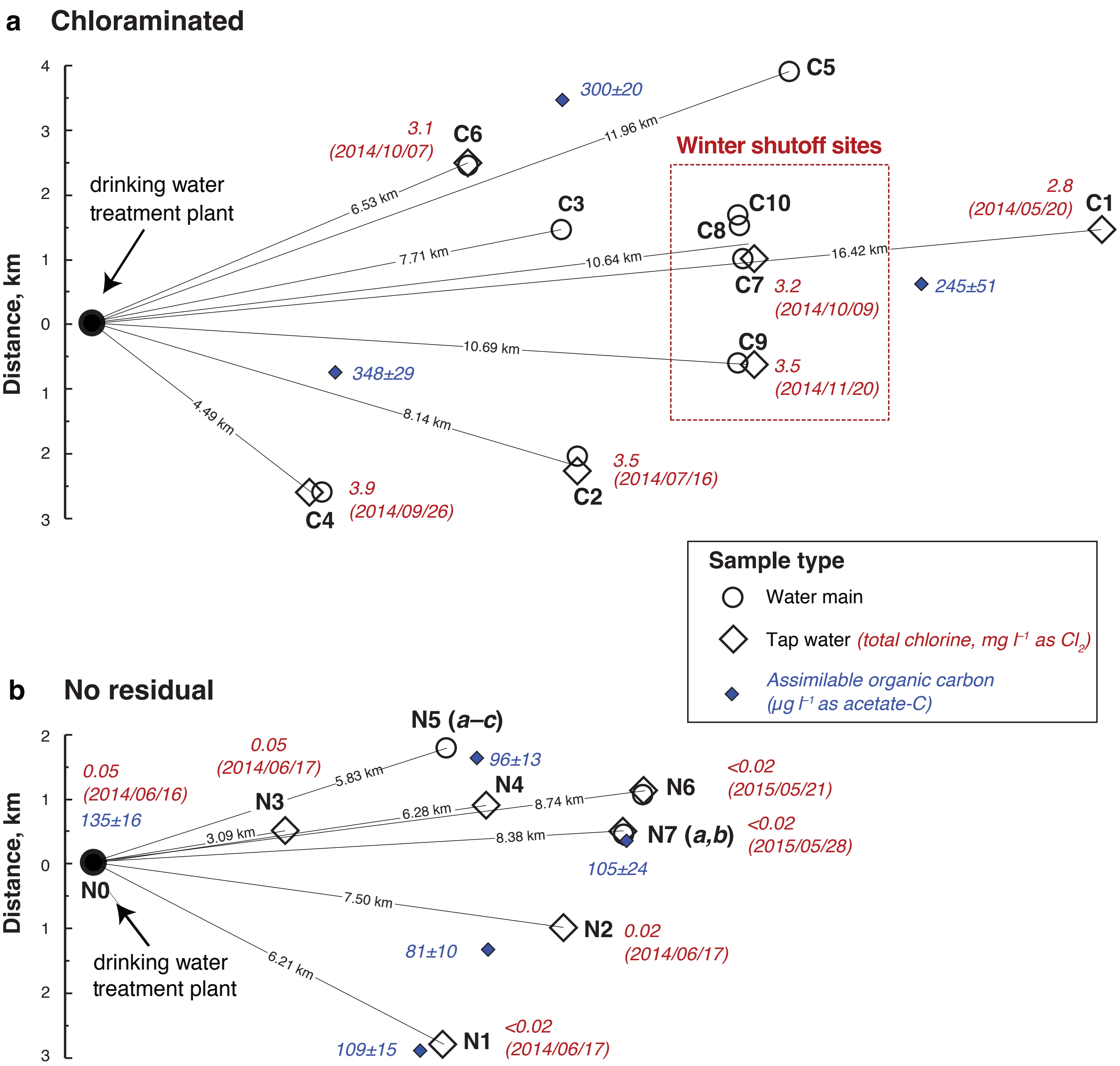

Figure S1. Locations of sample collection sites, with linear geographic distances relative to the drinking water treatment plants in the (**a**) chloraminated and (**b**) no-residual drinking water distribution systems in the United States and Norway, respectively. Total chlorine and assimilable organic carbon concentrations in drinking water are labelled near the respective measurement sites. Geographic scale between **a** and **b** are 1:1; cardinal directions and geographic features have been masked to retain anonymity of the two participating municipalities.

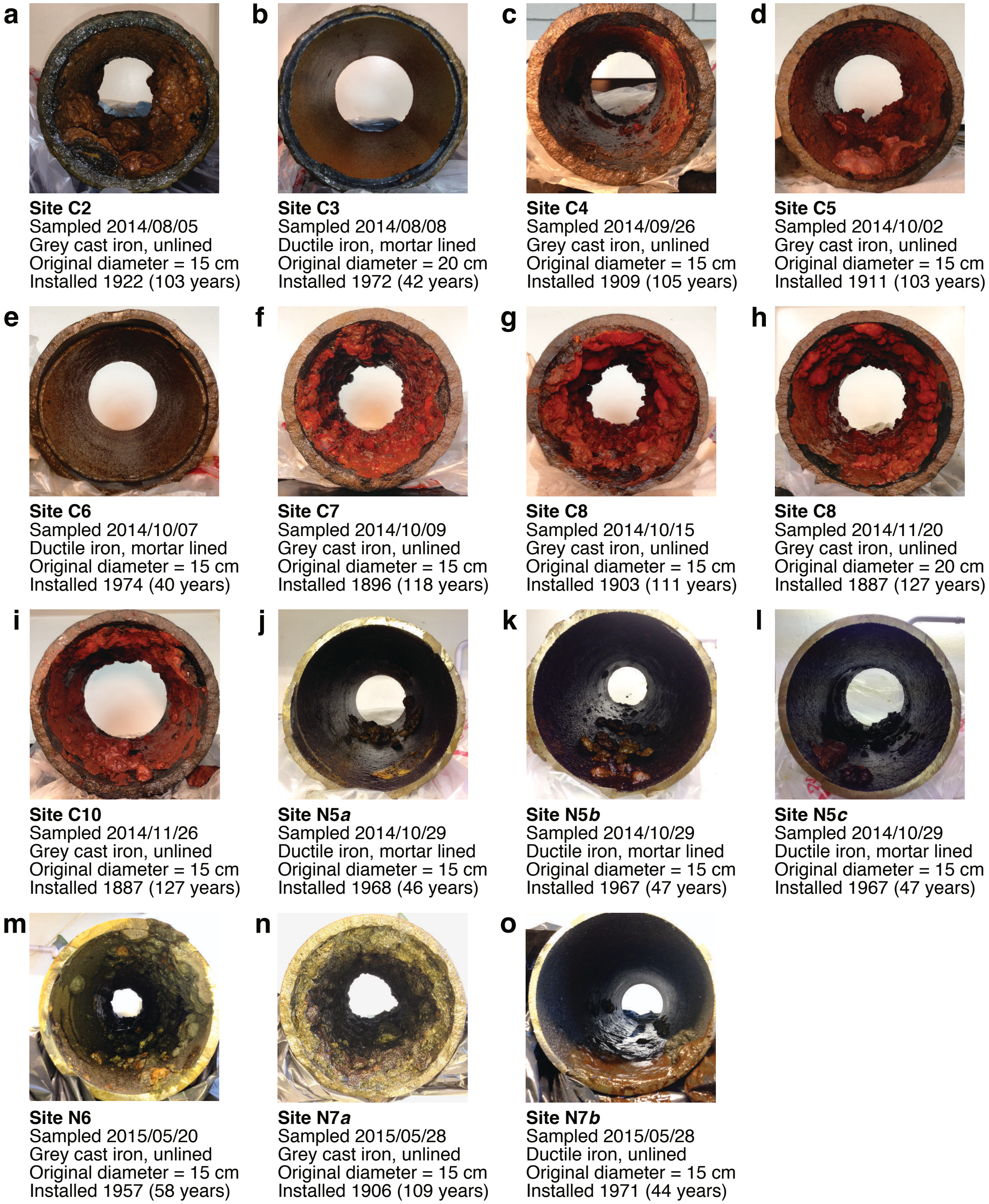

Figure S2. Water mains collected from the chloraminated (**a–i**) and no-residual (**j–o**) drinking water distribution systems.

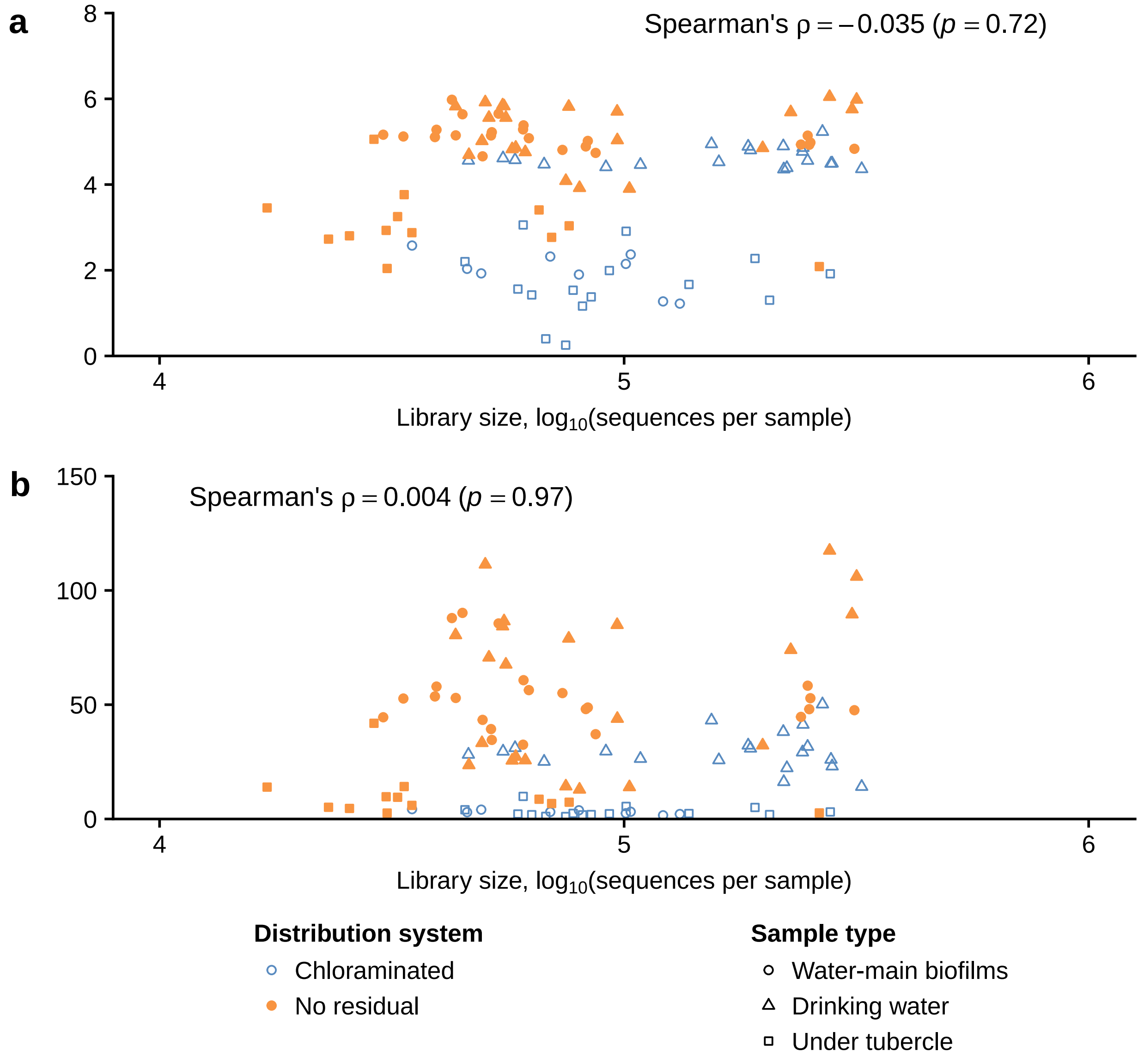

Figure S3. Within-sample (i.e., alpha) diversity as a function of library size among all samples in two drinking water distribution systems using the (**a**) Shannon index and (**b**) inverse Simpson index.

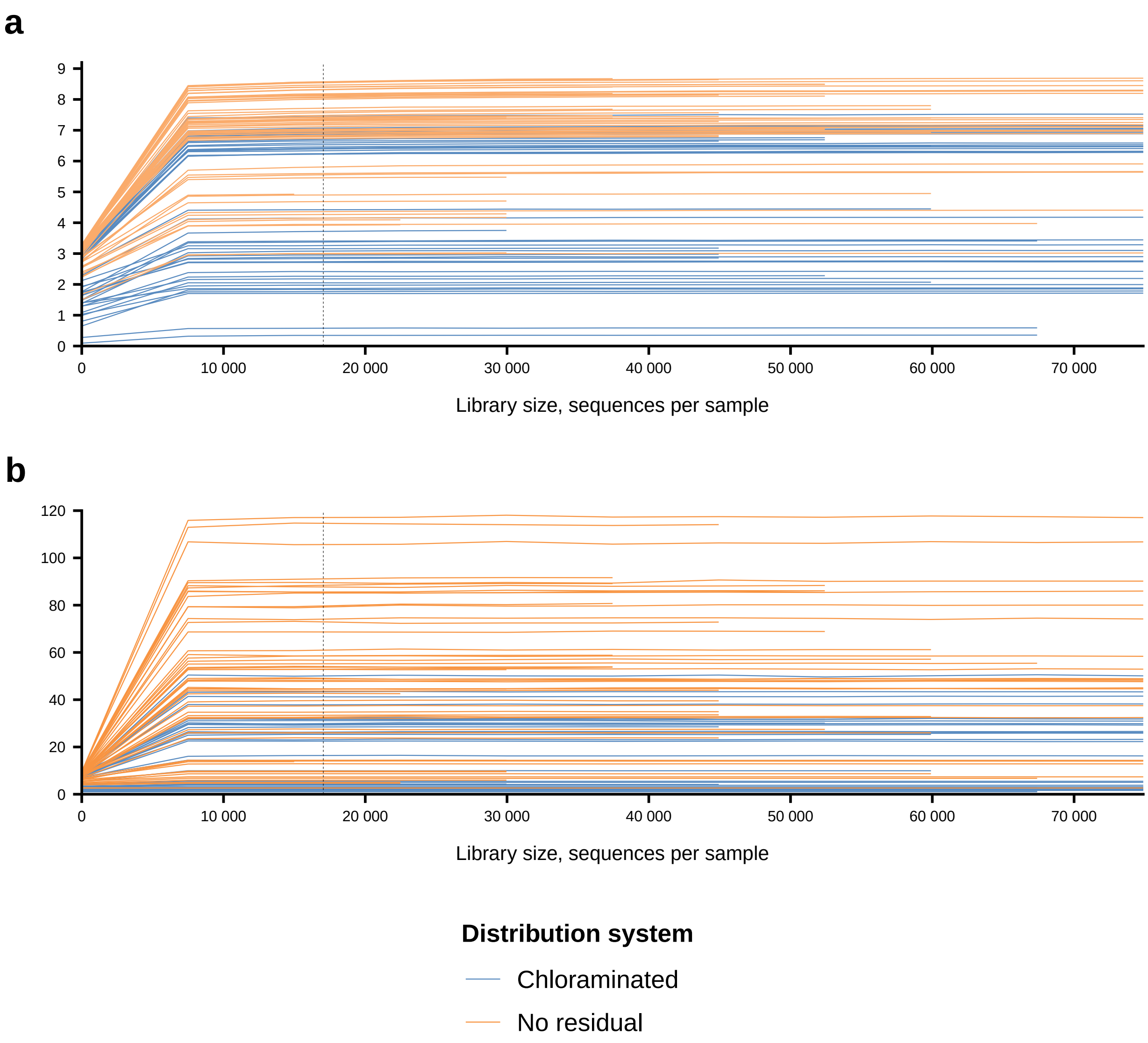

Figure S4. Rarefaction curves of within-sample (i.e., alpha) diversity as a function of varying library size among all samples in two drinking water distribution systems using the (**a**) Shannon index and (**b**) inverse Simpson index. Indices represent mean average of 10 random subsamples (without replacement). Subsamples were performed at 10 intervals within the range of 10 to 74 880 sequences per sample (i.e., the median library size). Dashed vertical lines indicate the minimum library size among all samples (17 041 sequences per sample); some samples terminate before this threshold due to the interval size of the rarefaction analysis.

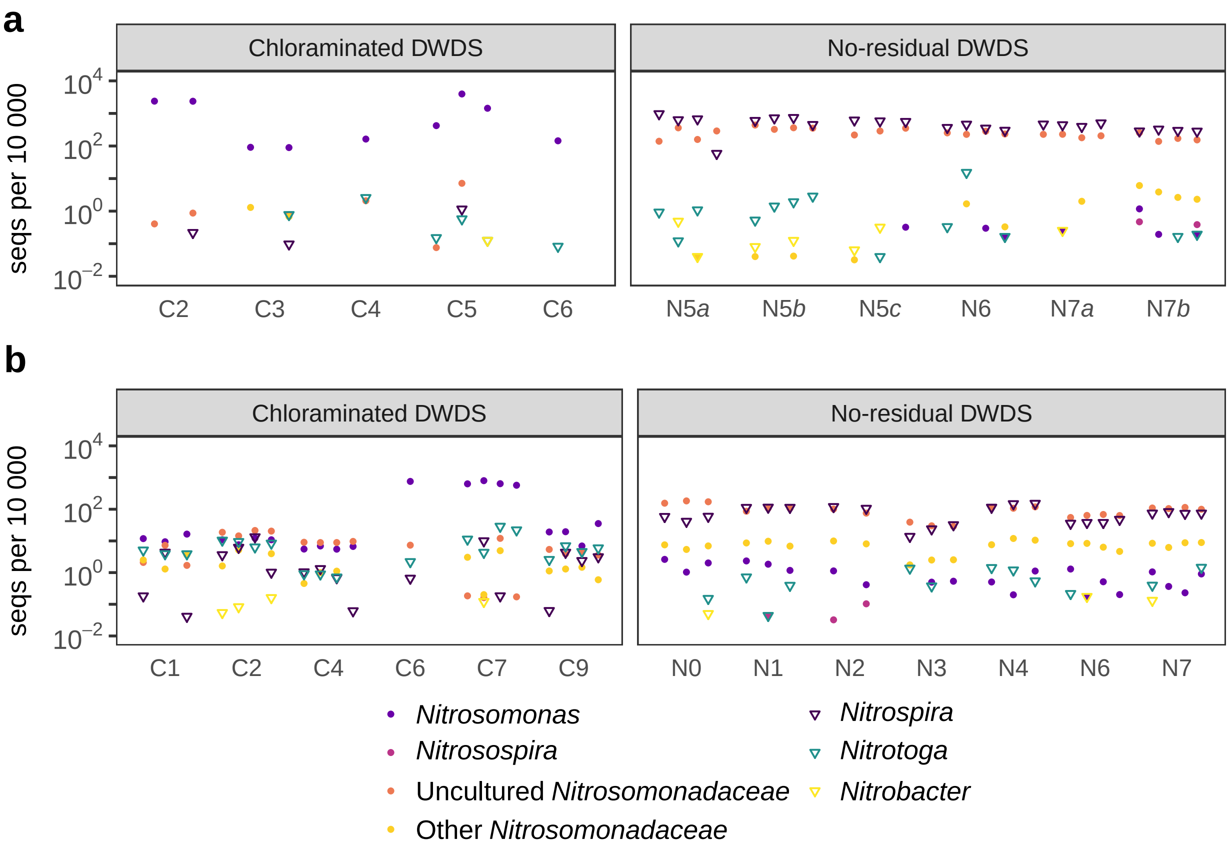

Figure S5. 16S rRNA gene sequence profiles of bacterial genera associated with ammonia oxidation (●) and nitrite oxidation (∇) in (**a**) water-main biofilms and (**b**) drinking water.

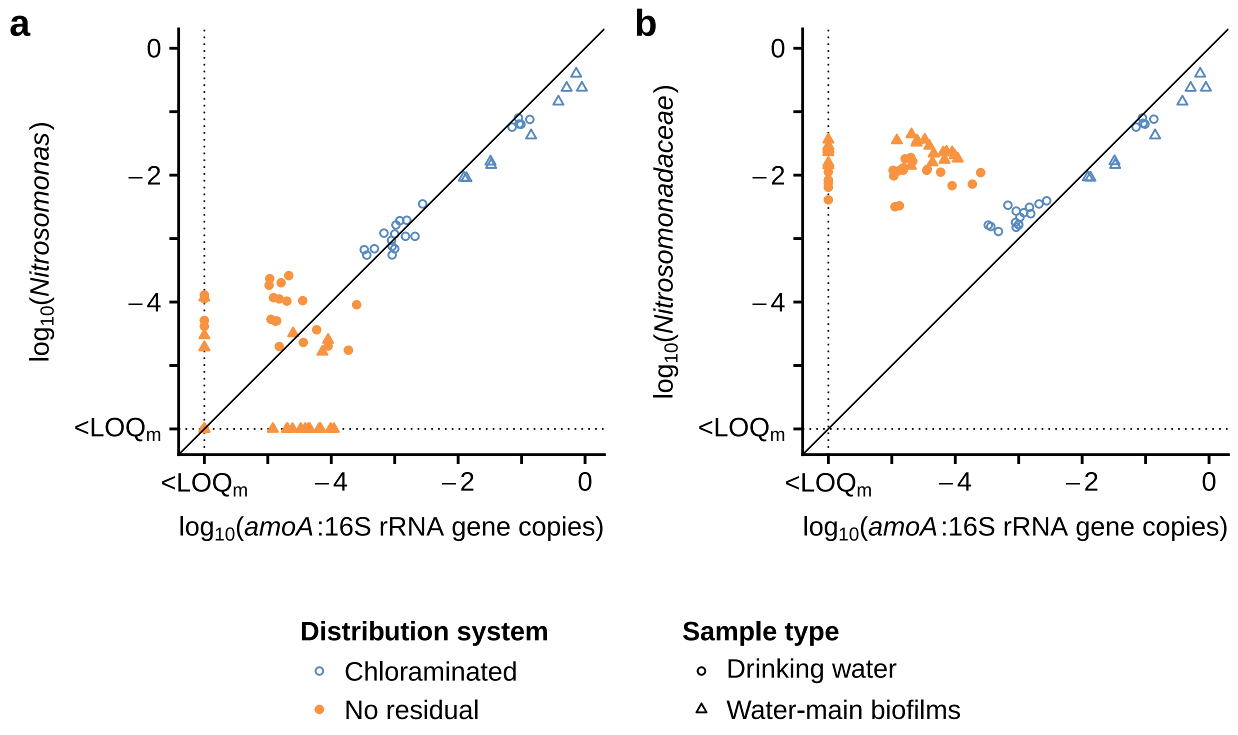

Figure S6. Relative abundances of operational taxonomic units (OTUs) from potential ammonia-oxidizing bacteria (AOB) versus *Nitrosomonas oligotropha*-like *amoA*:16S rRNA gene ratios: (**a**) genus *Nitrosomonas*-like OTUs and (**b**) family *Nitrosomonadaceae*-like OTUs.

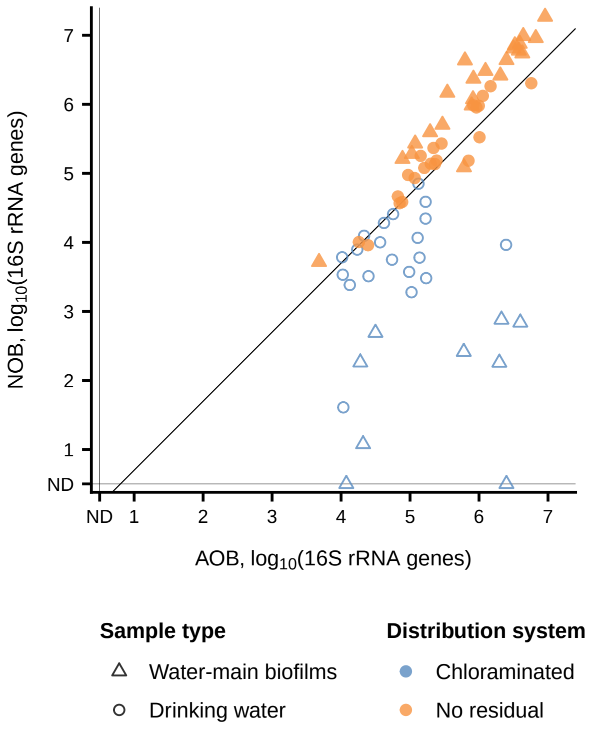

Figure S7. Comparison of nitrite-oxidizing bacteria (NOB) versus ammonia-oxidizing bacteria (AOB). Relative abundances of NOB-like operational taxonomic units (OTUs) (i.e., genera *Nitrospira*, *Nitrotoga*, and *Nitrobacter*) and AOB-like OTUs (family *Nitrosomonadaceae*) were normalized by 16S rRNA gene concentrations (via qPCR). The genera *Nitrosococcus*, *Nitrospinae*, *Nitrococcus*, *Nitrolancea*, and *Nitromaritima* were also considered but not observed. The theoretical NOB:AOB ratio for canonical nitrification is approximately 0.5. ND = no detection of specified taxa.

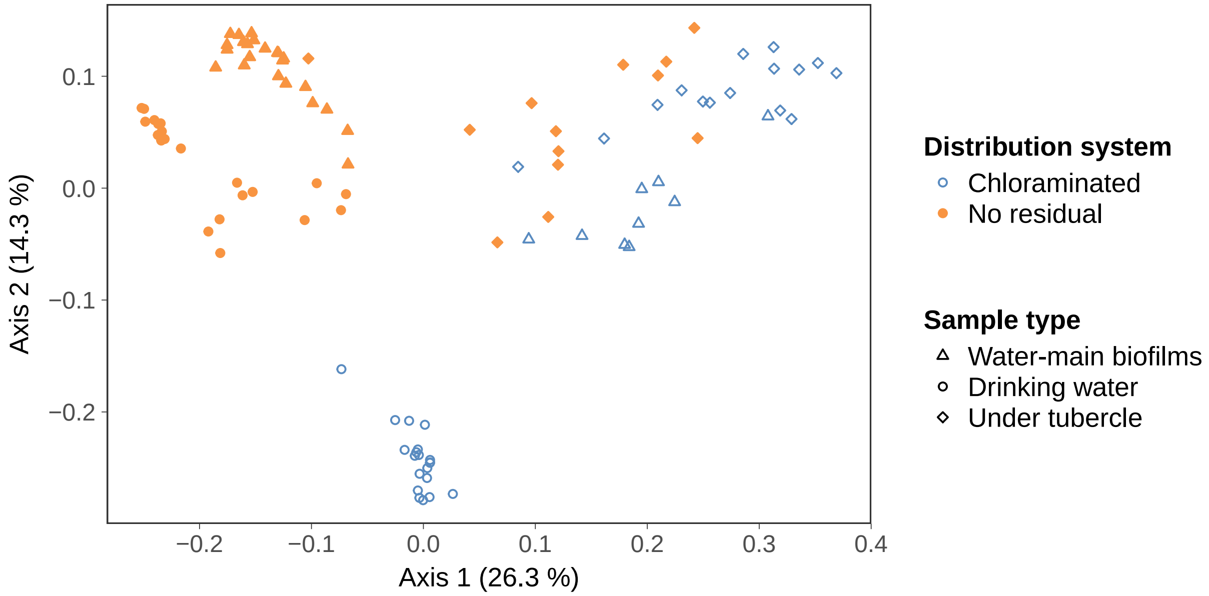

Figure S8. Principal coordinates analysis of generalized UniFrac for all samples collected from the chloraminated and no-residual drinking water distributions systems. This represents the between-sample (i.e., beta) diversity for water-main biofilms, drinking water, and under tubercle. Percentages = variance explained.

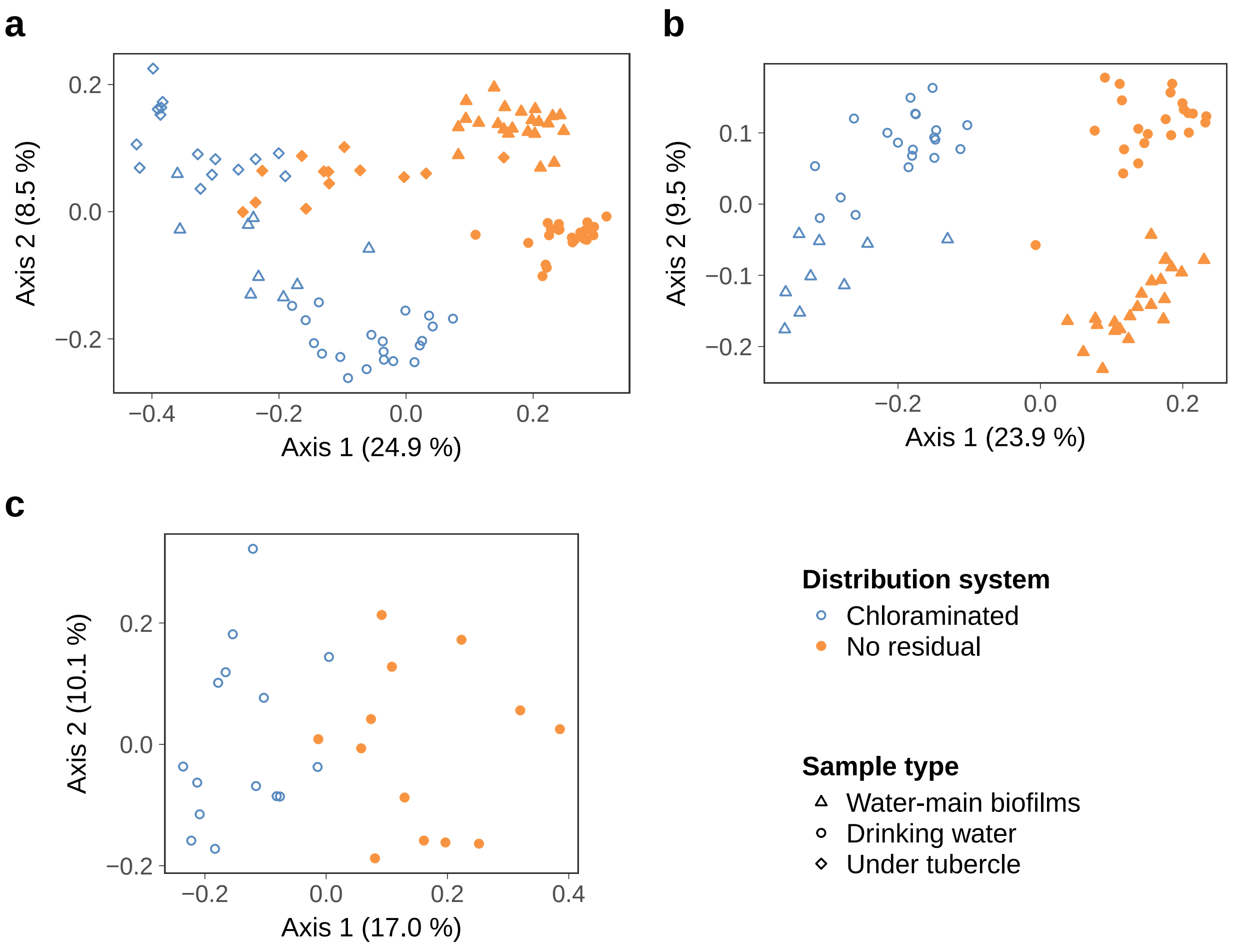

Figure S9. Principal coordinates analysis of unweighted UniFrac for samples collected from the chloraminated and no-residual drinking water distributions systems: (**a**) all samples, (**b**) water-main biofilm and drinking water only, and (**c**) under tubercle only. Percentages = variance explained.

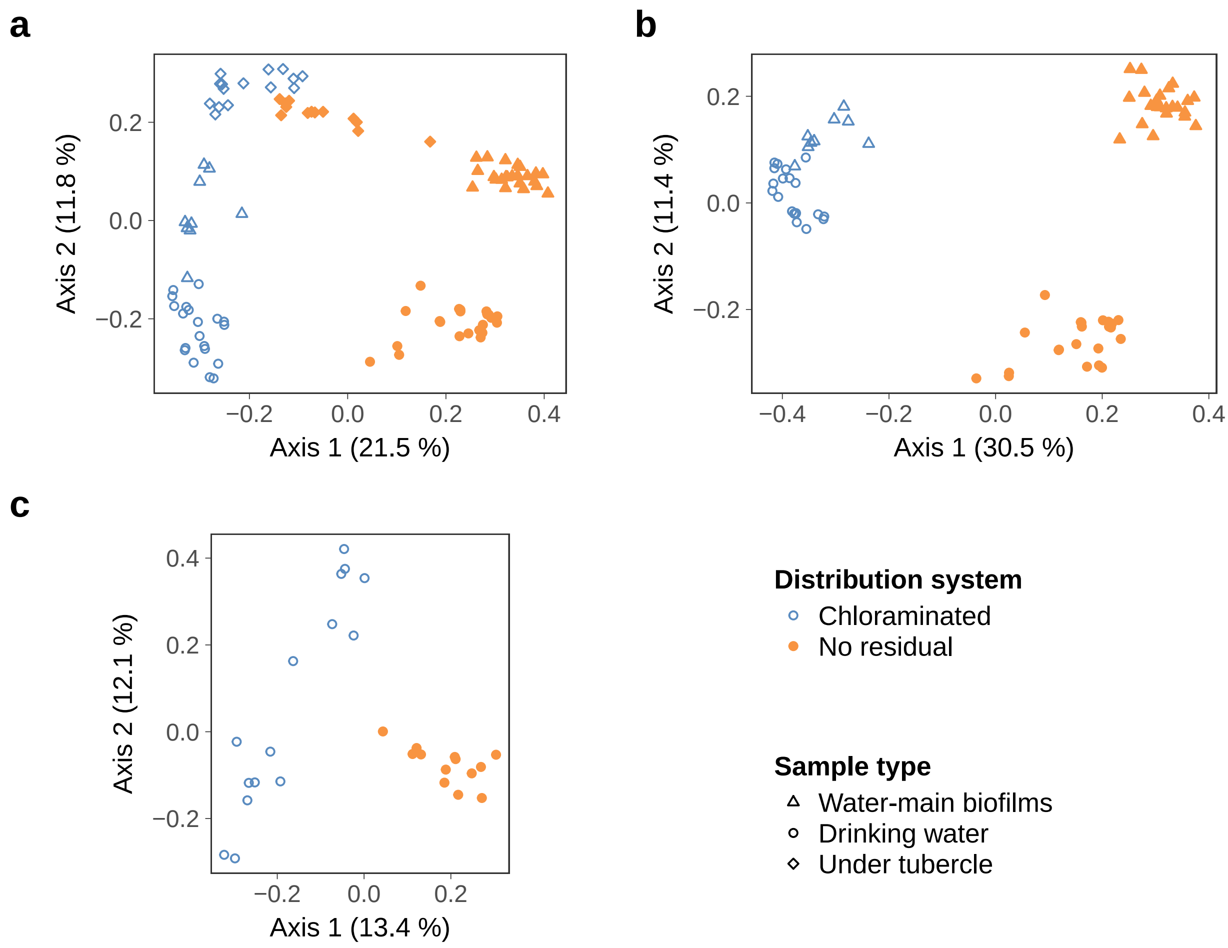

Figure S10. Principal coordinates analysis of Bray-Curtis dissimilarity for samples collected from the chloraminated and no-residual drinking water distributions systems: (**a**) all samples, (**b**) water-main biofilm and drinking water only, and (**c**) under tubercle only. Percentages = variance explained.

Table S1. Effect of library size on beta diversity metrics using different normalization methods[*]

	all samples		biofilm vs. water		under tubercle	
Metric[†]	R^2	P value	R^2	P value	R^2	P value
**Generalized UniFrac**						
No normalization	0.0320	0.001	0.0450	0.001	0.0757	0.016
Cumulative sum scaling	0.0357	0.001	0.0522	0.001	0.0670	0.012
Subsampling[‡]	0.0297	0.001	0.0430	0.001	0.0754	0.023
**Unweighted UniFrac**						
No normalization	0.0496	0.001	0.0618	0.001	0.0506	0.037
Cumulative sum scaling	0.0330	0.001	0.0618	0.001	0.0572	0.015
Subsampling[‡]	0.0169	0.012	0.0306	0.002	0.0513	0.046
**Bray-Curtis dissimilarity**						
No normalization	0.0643	0.001	0.1060	0.001	0.0877	0.004
Cumulative sum scaling	0.0389	0.001	0.0575	0.001	0.0557	0.004
Subsampling[‡]	0.0323	0.001	0.0505	0.001	0.0479	0.133

[*] Coefficients of determination (R^2) and P values were permuted (n = 999) with permutational multivariate analysis of variance (PERMANOVA, via the *adonis* function in the 'vegan' package)

[†] *Italics* indicates the final normalization method used for a metric (i.e., non-italicized methods are provided only for comparison)

[‡] Evenly subsampled library sizes (without replacement), set equal to the minimum sequence depth among included samples ('all samples' and 'under tubercle' = 17 041 sequences per sample; 'biofilm vs. water' = 30 304 sequences per sample)

Table S2. PCR primer sequences and thermoprofiles

Target	Primer name and sequence ($5' \rightarrow 3'$)		Size, bp	PCR thermoprofile	Reference
Bacterial 16S rRNA genes, V3	341F:<br>534R:	CCT ACG GGA GGC AGC AG<br>ATT ACC GCG GCT GCT GG	~ 200	1 min at 95 °C; 30 cycles of 15 s at 95 °C and 1 min at 60 °C	[S5]
Nitrosomonas oligotropha-like *amoA*	amo550F:<br>amo754R:	TCA GTA GCY GAC TAC ACM GG<br>CTT TAA CAT AGT AGA AAG CGG	205	1 min at 95 °C; 40 cycles of 15 s at 95 °C and 1 min at 56 °C	[S6]
Archaeal *amoA*	GenAOAF:<br>GenAOAR:	ATA GAG CCT CAA GTA GGA AAG TTC TA<br>CCA AGC GGC CAT CCA GCT GTA TGT CC	135	10 min at 95 °C; 40 cycles of 15 s at 95 °C and 30 s at 55 °C	[S7]

Table S3. Summary of real-time quantitative PCR reactions*

Target	LOQ, copy number	Amplification efficiency, %	R^2	Slope	Intercept
Nitrosomonas oligotropha-like *amoA*	5	97.2	0.994	–3.39	38.37
	5	99.7	0.990	–3.33	38.26
	5	93.0	0.994	–3.50	38.03
Archaeal *amoA*	130	94.4	0.999	–3.46	41.26
	130	96.6	0.999	–3.41	41.04
	130	94.8	0.996	–3.45	41.26

* LOQ, limit of quantification

Table S4. *P* values for *post hoc* group-wise comparisons of Shannon indices using the Conover-Iman test[*,†]

Kruskal-Wallis test $\boldsymbol{P = 2 \times 10^{-15}}$		water-main biofilm		drinking water		under tubercle	
		chlor.	no res.	chlor.	no res.	chlor.	no res.
water-main biofilm	chlor.	1	–	–	–	–	–
	no res.	<**0.0001**	1	–	–	–	–
drinking water	chlor.	<**0.0001**	<**0.0001**	1	–	–	–
	no res.	<**0.0001**	0.7852	<**0.0001**	1	–	–
under tubercle	chlor.	0.6285	<**0.0001**	<**0.0001**	<**0.0001**	1	–
	no res.	**0.0104**	<**0.0001**	**0.0001**	<**0.0001**	**0.0006**	1

[*] Chlor., chloraminated drinking water distribution system; no res., no-residual drinking water distribution system
[†] **Bold** indicates significance ($P \leq 0.05$)

Table S5. *P* values for *post hoc* group-wise comparisons of inverse Simpson indices using the Conover-Iman test[*,†]

Kruskal-Wallis test $P = 3 \times 10^{-15}$		water-main biofilm		drinking water		under tubercle	
		chlor.	no res.	chlor.	no res.	chlor.	no res.
water-main biofilm	chlor.	1	–	–	–	–	–
	no res.	<**0.0001**	1	–	–	–	–
drinking water	chlor.	<**0.0001**	<**0.0001**	1	–	–	–
	no res.	<**0.0001**	0.2878	<**0.0001**	1	–	–
under tubercle	chlor.	0.6253	<**0.0001**	<**0.0001**	<**0.0001**	1	–
	no res.	**0.0180**	<**0.0001**	<**0.0001**	<**0.0001**	**0.0014**	1

[*] Chlor., chloraminated drinking water distribution system; no res., no-residual drinking water distribution system
[†] **Bold** indicates significance ($P \leq 0.05$)
